# Enhancing the long-term abstinence rate of smoking through telemedicine: A multicenter, retrospective cohort study in Japan

**DOI:** 10.1371/journal.pone.0331514

**Published:** 2026-07-22

**Authors:** Kazuki Takakura, Tetsuro Ishizawa, Yoshihiko Aoki, Kei Matsumoto, Toshikazu D. Tanaka, Muneyuki Koyama, Yuji Kinoshita, Saburo Ito, Kazuhiro Yoshiuchi, Machi Suka

**Affiliations:** 1 Department of Internal Medicine, UnMed Clinic Motomachi, Yokohama, Kanagawa, Japan; 2 Department of Public Health and Environmental Medicine, The Jikei University School of Medicine, Tokyo, Japan; 3 Department of Stress Sciences and Psychosomatic Medicine, Graduate School of Medicine, The University of Tokyo, Tokyo, Japan; 4 Department of Gastroenterology and Hepatology, National Center for Global Health and Medicine Kohnodai Hospital, Chiba, Japan; 5 Division of Nephrology and Hypertension, Department of Internal Medicine, The Jikei University School of Medicine, Tokyo, Japan; 6 Division of Cardiology, Department of Internal Medicine, The Jikei University School of Medicine, Tokyo, Japan; 7 Department of Surgery, The Jikei University School of Medicine, Tokyo, Japan; 8 Division of Gastroenterology and Hepatology, Department of Internal Medicine, The Jikei University School of Medicine, Tokyo, Japan; 9 Division of Respiratory Diseases, Department of Internal Medicine, The Jikei University School of Medicine, Tokyo, Japan; King Abdulaziz University Faculty of Medicine, SAUDI ARABIA

## Abstract

The aim of this study is to analyze the differences between telemedicine and face-to-face examinations for smoking cessation in terms of long-term abstinence and completion rates in a large Japanese population. Although the effectiveness of various smoking cessation methods has been confirmed, the treatment outcomes of face-to-face interviews for tobacco cessation are still insufficient, because it is bother to go to a clinic regularly for most patients. In line with current trends of developing digital healthcare services, we assessed the role of telemedicine as a potential tool in smoking cessation clinics. A total of 5005 current smokers who applied for the telemedicine smoking cessation program between April 2021 and December 2022 and a total of 1308 current smokers who visited some smoking cessation clinics between April 2016 and March 2017 were included. Clinical data, such as age, sex, Brinkman index, were collected from all the participants, in addition to a preliminary questionnaire including smoking habits, reason for applying and self-efficacy for smoking cessation. This online program consists of four sessions in the first 8 weeks, with a 1-year follow-up period (final follow-up at 52 weeks). The success rates of long-term smoking abstinence at week 52 and the completion rates of scheduled treatment with varenicline or nicotine patches were compared between the telemedicine and face-to-face examinations. Data on face-to-face examinations were obtained from the database of the Ministry of Health, Labor and Welfare in Japan for comparison. A total of 3708 patients (3364 men and 352 women) were analyzed. The completion rate of this program (68.2%) was significantly greater than that of face-to-face examinations (29.8%). The long-term abstinence rate was significantly greater with telemedicine than with conventional examination (P < 0.005). In conclusion, the findings revealed that telemedicine is helpful for long-term abstinence from smoking on the basis of improved availability and accessibility.

## Introduction

Although the prevalence of tobacco consumption has been gradually decreasing worldwide, the Japanese smoking rate was still 20.1% in 2020, which was almost the same as the earlier rate [1]. Besides, the economic and social disparity in smoking has been expanding in Japan, similar to other developed countries, especially among the working generation [2]. In addition, the success rate of continuous smoking cessation is unsatisfactory because of the limited treatment persistence rate with conventional, face-to-face examination for smoking cessation [3,4]. In current situation of overcrowded medical facilities in Japan, patients have to wait for a long time to be seen by the doctor. It makes patients feel difficulty in receiving the medication for quitting smoking. However, a wide variety of evidence demonstrating the physical, mental, financial and social benefits of smoking cessation have been accumulating [5–15]. Considering not only the various health impairments stemming from smoking, but also the fact that tobacco is the primary cause of death in Japan [16], further promotion of smoking cessation and drastic measures for remedying smoking-related health inequalities at a younger age are urgently needed.

During the COVID-19 pandemic, the availability and accessibility of telemedicine, a digital health service, was promoted in various healthcare fields, including tobacco cessation clinics [17–19]. Indeed, several studies have reported the usefulness of telemedicine for smoking cessation, though limited number of patients or limited follow-up duration [20,21]. Since a previous study that used the same fully online smoking cessation program mainly focused on comparison of therapeutic outcomes between conventional cigarettes and heated tobacco products (HTPs) through telemedicine [22], we focused on comparing smoking cessation outcomes with telemedicine versus face-to-face examinations from a different perspective. Nicotine-dependent patients are commonly eligible for a 12-week standard smoking cessation treatment program with 5 clinic visits and an initial screening interview in which their exhaled carbon monoxide levels are checked in Japan [23]. Therefore, the duration of this smoking cessation program with varenicline was 8 weeks in a relatively short span of time, whereas that of the conventional program is 12 weeks with varenicline.

Given the insufficient clinical evidence regarding the usefulness of telemedicine for smoking cessation, we evaluated long-term smoking abstinence rates at 52 weeks from starting the program, as well as the completion rate of the scheduled treatment for 8 weeks in comparison with face-to-face examinations in this multicenter study involving thousands of participants.

## Materials and methods

### Study design and population

**Fig 1:**
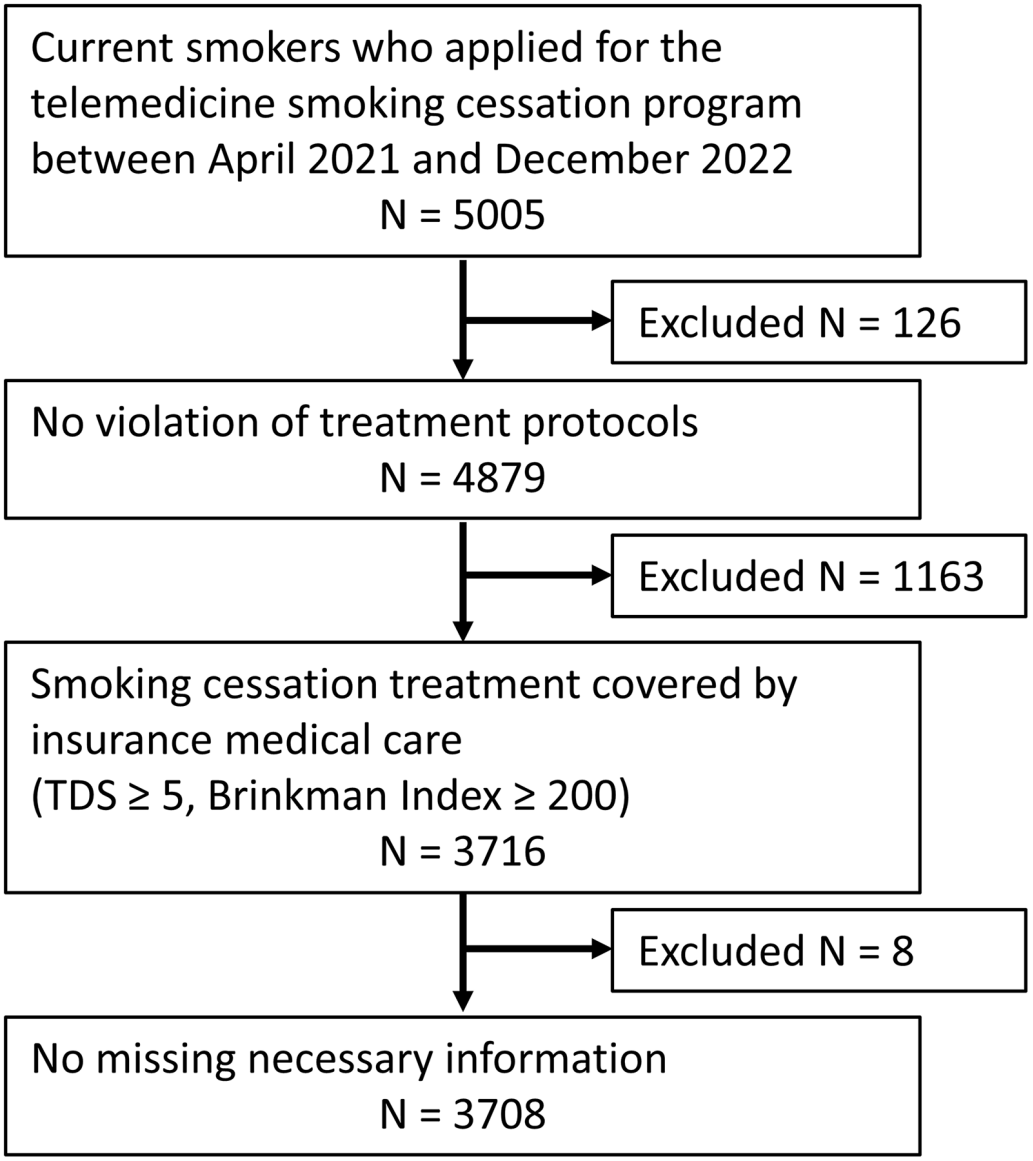
Diagram of the patients who applied for the telemedicine smoking cessation program in this study. Reasons for screening failure and discontinuation from the study are indicated.

A total of 5005 current smokers aged 20 years or older who applied for the telemedicine smoking cessation program through the health insurance association between 01/04/2021 and 31/12/2022 were potentially eligible for inclusion in this retrospective cohort study. Following enrollment into the cohort, the participants’ clinical information were accessed on 01/06/2024. A total of 20 Japanese medical institutes were entered, and the number of patients enrolled were at minimum 3, and maximum 1265. Most patients were participated not as an individual but from each business site. In a preliminary questionnaire, participants were asked about their duration of smoking, previous smoking cessation history, number of cigarettes per day, reason for applying to the program past medical history and self-efficacy for quitting smoking. Clinical data, such as age, sex, body mass index (BMI), Brinkman index, the Tobacco Dependence Screener (TDS) score [24] and treatment methods were collected at baseline and in follow-up surveys. In addition, the individual’s occupation, type of cigarette, drinking habits and complications of depressive symptoms identified by Whooley Questions for depression screening were also similarly recorded [25]. The degree of self-efficacy for quitting smoking was assessed by dividing the participants into 3 groups: low (0−24%), medium (25−74%) and high (75−100%). For the evaluation of therapeutic efficacy, 1297 censored patients were finally excluded from the analyses, as shown in Fig 1. A summary of the data collected from the study patients is presented in Table 1. The study protocol was approved by the ethics committee of UnMed Clinic Motomachi, Kanagawa, Japan (authorization number: UM24−01). The review board approved and waived the need for written informed consent from the participants because of the retrospective, noninterventional nature of this study. The study followed the recommended guidelines of the Declaration of Helsinki.

**Table 1:** Demographic and clinical characteristics of the study participants.

Parameters	Value
Age, mean (SD), years	43.6 (10.5)
Sex, n (%)	
Men	3356 (90.5)
Women	352 (9.5)
Body Mass Index, n (%) ≥ 25	1276 (34.4)
Alcohol habits, n (%) 4 days and above in a week	1302 (35.1)
Occupation, n (%)	
White-collar worker	2201 (59.4)
Blue-collar worker	1507 (40.6)
Cigarettes type, n (%)	
Combustible cigarette	1499 (40.4)
Heated tobacco products	1582 (42.7)
Dual smoking	627 (16.9)
Electronic cigarette combined	798 (21.5)
Number of cigarettes per day, n (%)	54.4 (41.1)
1-20	3268 (88.1)
21-40	366 (9.9)
> 40	74 (2.0)
Smoking years, n (%)	
1-20	1536 (41.4)
21-40	1916 (51.7)
> 40	256 (6.9)
Brinkman index, n (%)	
1-399	1729 (46.6)
400-699	1376 (37.1)
≥ 700	603 (16.3)
Tobacco Dependence Screener score, n (%)	
5-6	1046 (28.2)
7-8	1720 (46.4)
9-10	942 (25.4)
Past quit attempt, n (%)	
yes	2455 (66.2)
no	1253 (33.8)
Secondhand smoking, n (%)	1536 (41.4)
Self-efficacy, n (%)	
0-24	796 (21.5)
25-49	502 (13.5)
50-74	1821 (49.1)
≥ 75	589 (15.9)

A summary of qualitative and quantitative variables in the study.

### The telemedicine smoking cessation program

**Fig 2:**
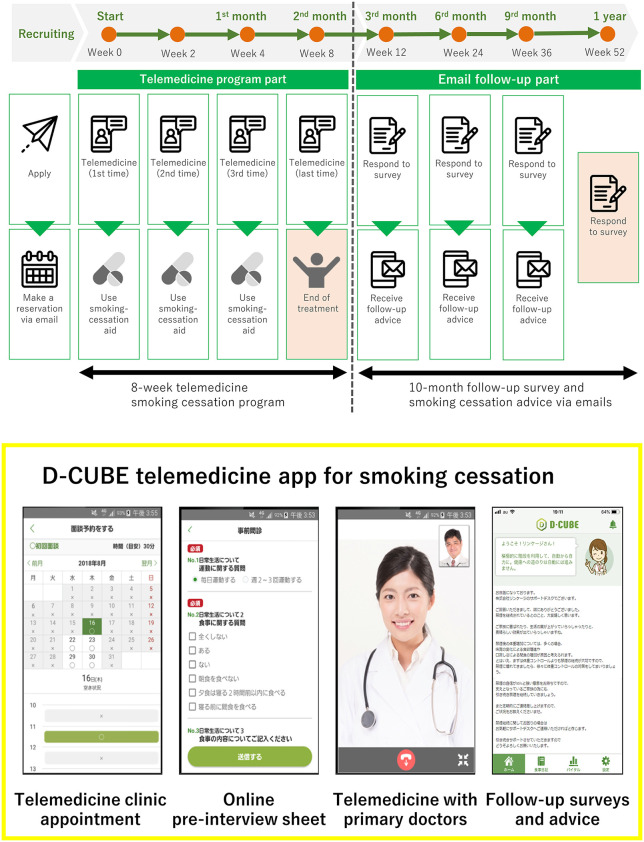
Overview of the Linkage telemedicine smoking cessation program and the image of telemedicine app.

The smoking cessation program, provided online by Linkage, Tokyo, Japan, was implemented at all the clinics that participated in this study (Fig 2) [22]. This fully remote online program consists of four sessions in the first 8 weeks, with a total follow-up period of 1 year via surveys and support via an app at weeks 12, 24, 36 and 52. In the telemedicine sessions, the doctors assessed the participants’ progress in tobacco cessation and provided practical advice on quitting smoking each time. Varenicline or nicotine patches were selectively prescribed as anti-smoking drugs on the basis of the patient’s condition. The participants could receive varenicline or nicotine patches directly at home for a total duration of 8 weeks without the need for clinic visits. Moreover, qualified experts of smoking cessation provided constant supports for the patients via email within the follow-up period.

### Conventional smoking cessation program

Conventional program consists of five face-to-face examinations in the first 12 weeks, with a total follow-up period of 52 weeks. The patients selectively received varenicline for a total duration of 12 weeks or nicotine patches for a total duration of 8 weeks at each medical institutes. Data on conventional therapy were obtained from the most recent database of the Ministry of Health, Labor and Welfare (MHLW) in Japan for comparison of this study. A total of 1308 current smokers who visited some smoking cessation clinics between April 2016 and March 2017 were included in the database reported in 2017 [23]. A total treatment duration with varenicline (12 weeks) is 4 weeks longer than that with the telemedicine program (8 weeks), although a total treatment duration with nicotine patches (8 weeks) and total follow-up period of 52 weeks are the same.

### Outcomes

The primary outcome was the long-term smoking abstinence rate at week 52 after 8 weeks of treatment with varenicline or nicotine patches following treatment through telemedicine or face-to-face interviews and its related factors.

The secondary outcome was completion rates of the scheduled treatment in the context of telemedicine versus face-to-face interviews and its related factors. Additionally, we compared the therapeutic efficacy of varenicline versus nicotine patches, a form of nicotine replacement therapy (NRT), administered through the online program**.**

### Statistical analysis

All the statistical analyses were performed using SAS V.9.4 software (SAS Institute, Cary, NC, USA). The percentages of patients who completed the scheduled treatment with varenicline and nicotine patches were compared using the χ-square test. Completion rates were compared with those reported by the MHLW using the one-sample Z-test. Multiple logistic regression analysis was performed to identify factors associated with treatment completion. Odds ratios (ORs) and 95% confidence intervals (CIs) for treatment completion were calculated using multivariate adjustment. Independent variables to be input into the model were confirmed that there is no strong correlation among them (r > 0.80). The result of Hosmer-Lemeshow goodness-of-fit test was good (p = 0.898). The significance level was set at p < 0.05.

## Results

### Participant characteristics

A total of 3708 patients who participated in the smoking cessation program at several medical institutes between April 2021 and December 2022 were included in the analyses (Fig 1). The baseline characteristics of the study participants are summarized in Table 1. The median age of the enrolled patients was 43.6 ± 10.5 years, with 3364 (90.5%) men and 352 (9.5%) women. Among them, 65.5% were underweight or of normal weight (BMI < 25 kg/m^2^), and 34.4% were overweight (BMI ≥ 25 kg/m^2^). The types of smoking products included combustible cigarettes in 40.4%, and HTP in 42.7%, while 16.9% were dual users. Although the number of cigarettes smoked was < 20 per day in 88.1% of the participants, the duration of smoking ranged from 1–20 years in 41.5%, 21–40 years in 51.6% and more than 40 years in 6.9%. Alcohol was consumed by 1694 participants (45.6%), and 987 (26.6%) had mental illnesses. In terms of occupation, blue- and white-collar workers numbered 1507 (40.6%) and 2201 (59.4%), respectively. According to the preliminary questionnaire, the degree of self-efficacy at the initiation of treatment was low in 21.5%, medium in 62.6% and high in 15.9% of the study participants.

A total of 1308 current smokers who visited some smoking cessation clinics between April 2016 and March 2017 were included for comparison. The most common age of the enrolled patients was 40–49 years, with 873 (66.7%) men, 400 (30.6%) women and 35 (2.7%) non-respondent patients.

### High completion rate of scheduled treatment through telemedicine

The completion rate of scheduled treatment in the fully online program (68.2%) was significantly higher than that of face-to-face examinations (34.6%) reported by the MHLW. This was possibly due to the easy accessibility of telemedicine without the need for clinic visits and the availability of direct home-delivery of the drugs. Moreover, varenicline (78.8%) showed significantly higher completion rates than nicotine patches (65.4%) in the online program, similar to conventional treatment (P < 0.001) (Table 2).

**Table 2:** The completion and abstinence rate of scheduled treatment for 8 weeks.

	n	Completion (n, %)		Abstinence (n, %)	
Total	3708	3411	92.0	2529	68.2
varenicline	805	747	92.8	634	78.8
nicotine patch	2893	2659	91.9	1893	65.4
None	10	5	50.00	2	20.0
			P = 0.411		P < 0.001

### Factors related to the completion rate according to logistic regression analysis

As shown in Table 3, older age (OR 1.99; 95% CI 1.40–2.82), HTP user (OR 1.37; 95% CI 1.02–1.84), a higher Brinkmann index (OR 1.07; 95% CI 1.00–1.15) and higher self-efficacy (OR 1.08; 95% CI 1.03–1.14) were significant factors in the completion rate of the scheduled treatment according to logistic regression analysis. Conversely, sex, BMI, alcohol habits, occupation, mental disorders, TDS score and non-smoker status were not significantly correlated with the completion rate of the scheduled treatment.

**Table 3:** Related factors for the completion rate of scheduled treatment.

Parameters	total	completion	%	p	odds ratios	95%CI
**Age**						
20-44	1840	1636	88.9	<0.001	1	
≥ 45	1858	1770	95.3		1.99	1.40-2.82
**Sex**						
Men	3347	3083	92.1	0.953	1	
Women	351	323	92.0		0.98	0.65-1.49
**Body Mass Index**						
< 25	2427	2237	92.2	0.833	1	
≥ 25	1271	1169	92.0		0.89	0.69-1.15
**Alcohol habits**						
within 0–3 days a week	2400	2203	91.8	0.339	1	
4 days and above in a week	1298	1203	92.7		0.92	0.70-1.20
**Occupation**						
White-collar worker	2194	2042	93.1	0.008	1	
Blue-collar worker	1504	1364	90.7		0.84	0.66-1.07
**Depressive symptoms**						
none	2718	2509	92.3	0.438	1	
yes	980	897	91.5		1.02	0.77-1.34
**Cigarettes type**						
Combustible cigarette	1495	1378	92.2	<0.001	1	
Heated tobacco products	1578	1478	93.7		1.37	1.02-1.84
Dual smoking	625	550	88		0.72	0.52-0.99
Electronic cigarette combined	795	734	92.3		1.05	0.77-1.44
**Brinkman index**						
1-399	1722	1542	89.5	<0.001	1.07	1.00-1.15
400-699	1375	1298	94.4			
≥ 700	601	566	94.2			
**Tobacco Dependence Screener score**						
5-6	1040	972	93.5	0.076	1	0.92-1.10
7-8	1719	1566	91.1			
9-10	939	869	92.4			
**Past quit attempt**						
none	1285	1177	91.6	0.403	1	
yes	2413	2229	92.4		1.02	0.79-1.31
**Self-efficacy**						
0-24	795	710	89.3	0.003	1.08	1.03-1.14
25-49	502	457	91.0			
50-74	1818	1694	93.2			
≥ 75	583	545	93.5			
**Drug**						
varenicline	805	747	92.8	0.411	1	
nicotine patch	2893	2659	91.9		0.95	0.70-1.29

### Telemedicine enhanced long-term smoking cessation rates

A total of 3698 patients were included in the comparative analysis of the results of the 1-year follow-up. Four hundred thirty-three of 805 varenicline users (53.8%) and 1230 out of 2893 nicotine patch users (42.5%) were able to quit smoking continuously, even though only 28.3% of the varenicline users and 34.5% of the nicotine patch users were in the same setting reported from the MHLW in Japan (Table 4). Taken together, the long-term abstinence rate was significantly greater with telemedicine rather with conventional examination regardless of the therapeutic method used (P < 0.001).

**Table 4:** The comparison of the long-term abstinence rate at 52 weeks.

Full online program	total	abstinence	%
varenicline	805	433	53.8
nocotine patch	2893	1230	42.5
**Face-to-face examination (data from MHLW)**			
varenicline	916	259	28.3
nicotine patch	145	50	34.5
**the difference in the population proportions**	Z	p	
varenicline	16.015	< 0.0001	
nicotine patch	9.051	< 0.0001	

MHLW: Ministry of Health, Labour and Welfare.

### Factors related to the abstinence rate according to logistic regression analysis

Next, we assessed factors related to the abstinence rate at the 1-year follow-up from therapeutic initiation. As shown in Table 5, older age (OR 1.60; 95% CI 1.34–1.91), blue-collar workers (OR 0.85; 95% CI 0.74–0.98), depressive symptoms (OR 0.82; 95% CI 0.71–0.96), HTP users (OR 1.34; 95% CI 1.15–1.56) and higher self-efficacy (OR 1.05; 95% CI 1.02–1.08) were significantly related to outcomes. In addition, participants who used varenicline had a higher abstinence rate than those who received nicotine patches in the online program (OR 0.66; 95% CI 0.57–0.78). On the other hand, sex, BMI, alcohol habit, Brinkman index, TDs score and non-smoking status were not correlated with the long-term abstinence rate.

**Table 5:** Related factors for the long-term abstinence rate at 52 weeks.

Parameters	odds ratios	95% confidence interval	p
**Age**			
20-44	1		
≥ 45	1.6	1.34-1.91	<0.001
**Sex**			
Men	1		
Women	0.94	0.83-1.16	0.578
**Body Mass Index**			
< 25	1		
≥ 25	1.04	0.91-1.20	0.550
**Alcohol habits**			
within 0–3 days a week	1		
4 days and above in a week	1.03	0.90-1.19	0.664
**Occupation**			
White-collar worker	1		
Blue-collar worker	0.85	0.74-0.98	0.025
**Depressive symptoms**			
none	1		
yes	0.82	0.71-0.96	0.014
**Cigarettes type**			
Combustible cigarette	1		
Heated tobacco products	1.34	1.15-1.56	<0.001
Dual smoking	0.73	0.60-0.90	0.003
Electronic cigarette combined	0.93	0.78-1.10	0.383
**Brinkman index**	1.02	0.99-1.05	0.297
**Tobacco Dependence Screener score**	0.98	0.93-1.02	0.320
**Past quit attempt**			
none	1		
yes	0.99	0.87-1.15	0.991
**Self-efficacy**			
0-24	1.05	1.02-1.08	<0.001
25-49			
50-74			
≥ 75			
**Drug**			
varenicline	1		
nicotine patch	0.66	0.57-0.78	<0.001

## Discussion

This multicenter, retrospective cohort study revealed significant difference in long-term smoking abstinence rates between the group treated through telemedicine and the group treated through face-to-face interviews at 52 weeks from treatment initiation. The improvement in the abstinence rates observed in the present study highlights the better completion rate of the scheduled treatment with the online program, likely since it is a more convenient therapeutic method and contributes to enhance self-efficacy for quitting smoking. Telemedicine allows smokers to undergo medical consultations and to receive prescriptions without the need for clinic visits, whereas a total of 5 times follow-up visits is needed in face-to-face examinations. Moreover, it is conceivable that the constant supports from our experts of smoking cessation within the 1-year follow-up also contributed for this online program. A previous report from Japan, which also compared the results of telemedicine versus conventional examinations, evaluated short-term abstinence rates for smoking cessation [26]. Another study from the United States assessed telemedicine versus telephone counseling for smoking abstinence at month 12 [20]. In addition, a report from Turkey showed the utility of telemedicine in comparison to traditional counseling by evaluating smoking cessation status at 6–9 months of follow-up [21]. To the best of our knowledge, this is the first large-scale study to compare the long-term smoking abstinence rates achieved by telemedicine versus face-to-face examination. Since the risk of smoking relapse is particularly high within the first year after cessation, we followed the patients for 1 year after treatment initiation in this study and evaluated the therapeutic outcomes at that time [27]. Thus, our results help elucidate the potential role of telemedicine in smoking cessation.

In this study, it is noteworthy that self-efficacy was a significant factor not only for the completion rate, but also for the abstinence rate. The results suggest that a telemedicine-supported program has a greater impact on improving self-efficacy for tobacco cessation, leading to better outcomes. The fact that self-efficacy is closely related to supporting smoking cessation is already well known [28–32]. Our data are consistent with previous studies and suggest that self-efficacy in quitting smoking can be positively influenced by the availability of treatment.

Interestingly, occupation, whether the participant was a blue-collar or white-collar worker, was a significant factor in the long-term abstinence rate, although there was no correlation with age. As previously reported, responding to socioeconomic inequalities in relation to smoking cessation still remains a task for the future [2,33]. Additionally, since several studies have indicated that younger smokers are more likely to be able to quit smoking successfully than older smokers are, further investigations into age-related factors for successful smoking cessation are needed [34,35].

In the comparison of varenicline and NRT in the online program, varenicline yielded better results than nicotine patch in terms of both the completion rate and the long-term abstinence rate, similar to the results for face-to-face examinations [36,37]. Although, varenicline is currently not readily available in Japan, the resumption of varenicline sales is expected very quickly [38].

This study has some limitations. First, the participants in this program were all members of the Japanese health insurance association, i.e., they were all within the working generation. Hence, it is likely that there is some selection bias in this study. Second, a total treatment duration with varenicline through this online program was 8 weeks, even though varenicline is usually prescribed for 12 weeks at conventional, face-to-face clinics [39–41]. For a valid comparison, these two programs should be similar to ensure that any differences are due to the delivery format rather than other factors. Thus, we compared the study data with pre-existing data reported from the MHLW by setting the same follow-up period of 52 weeks from treatment initiation, to unify the total follow-up period. Third, continuous abstinence rates were assessed by questionnaire-based self-reported abstinence, but not biochemically validated, which could misclassify smoking status or overestimate abstinence rates. Fourth, we excluded participants unavailable for follow-up surveys in the 52 weeks, which could potentially affect the completion and success rates of the telemedicine smoking cessation program. Finally, our data suggests differences in certain trends between white-collar worker and blue-collar worker. Thus, participant demographics, such as white-collar worker and blue-collar worker, may have an impact on the results of smoking cessation according to the differences in resources including working environment.

A key strength of our study was the large number of participants included from multiple medical institutions with complete follow-up for 52 weeks. Our study provides definitive insights into continuous smoking abstinence and the potential efficacy of telemedicine in smoking cessation.

In conclusion, telemedicine is definitely helpful for long-term abstinence from smoking, on the basis of its improved availability and accessibility. The higher completion rate of the scheduled treatment was likely a key player in the observed therapeutic outcomes. In addition, the resumption of varenicline sales is desirable, in view of its better outcomes than NRT in telemedicine, which is similar to conventional examinations.

## References

[1] WHO global report on trends in prevalence of tobacco use 2000–2030. Geneva: World Health Organization.2024.

[2] Tanaka H, Mackenbach JP, Kobayashi Y. Widening socioeconomic inequalities in smoking in Japan, 2001-2016. J Epidemiol. 2021;31(6):369–77. 32595181 10.2188/jea.JE20200025 PMC8126678

[3] Hughes JR, Peters EN, Naud S. Relapse to smoking after 1 year of abstinence: A meta-analysis. Addict Behav. 2008;33(12):1516–20. doi: 10.1016/j.addbeh.2008.05.012 18706769 PMC2577779

[4] Agboola SA, Coleman T, McNeill A, Leonardi-Bee J. Abstinence and relapse among smokers who use varenicline in a quit attempt-a pooled analysis of randomized controlled trials. Addiction. 2015;110(7):1182–93. doi: 10.1111/add.12941 25846123

[5] Khalifeh M, Ginex P, Boffetta P. Reduction of head and neck cancer risk following smoking cessation: A systematic review and meta-analysis. BMJ Open. 2024;14(8):e074723. doi: 10.1136/bmjopen-2023-074723 39122405 PMC11331934

[6] Ravidà A, Saleh MHA, Ghassib IH, Qazi M, Kumar PS, Wang H-L, et al. Impact of smoking on cost-effectiveness of 10-48 years of periodontal care. Periodontol 2000. 2025;98(1):32–44. doi: 10.1111/prd.12585 39054672 PMC12842896

[7] Ayaz D, Asi E, Meydanlioglu A, Oncel S. Effectiveness of smoking cessation interventions in the workplace: A systematic review and meta-analysis. Am J Ind Med. 2024;67(8):712–22. doi: 10.1002/ajim.23627 38884628

[8] Walicka M, Krysiński A, La Rosa GRM, Sun A, Campagna D, Di Ciaula A, et al. Influence of quitting smoking on diabetes-related complications: A scoping review with a systematic search strategy. Diabetes Metab Syndr. 2024;18(5):103044. doi: 10.1016/j.dsx.2024.103044 38810420

[9] Lai H, Liu Q, Ye Q, Liang Z, Long Z, Hu Y, et al. Impact of smoking cessation duration on lung cancer mortality: A systematic review and meta-analysis. Crit Rev Oncol Hematol. 2024;196:104323. doi: 10.1016/j.critrevonc.2024.104323 38462148

[10] Wahbeh F, Restifo D, Laws S, Pawar A, Parikh NS. Impact of tobacco smoking on disease-specific outcomes in common neurological disorders: A scoping review. J Clin Neurosci. 2024;122:10–8. doi: 10.1016/j.jocn.2024.02.013 38428126 PMC10978265

[11] Higashi Y. Smoking cessation and vascular endothelial function. Hypertens Res. 2023;46(12):2670–8. doi: 10.1038/s41440-023-01455-z 37828134 PMC10695829

[12] Halms T, Strasser M, Hasan A, Rüther T, Trepel M, Raab S, et al. Smoking and quality of life in lung cancer patients: Systematic review. BMJ Support Palliat Care. 2024;13(e3):e686–94. doi: 10.1136/spcare-2023-004256 37607808

[13] Delcroix M-H, Delcroix-Gomez C, Marquet P, Gauthier T, Thomas D, Aubard Y. Active or passive maternal smoking increases the risk of low birth weight or preterm delivery: Benefits of cessation and tobacco control policies. Tob Induc Dis. 2023;21:72. doi: 10.18332/tid/156854 37256119 PMC10226447

[14] Hoch JS, Barr HK, Guggenbickler AM, Dewa CS. Lessons from cost-effectiveness analysis of smoking cessation programs for cancer patients. Curr Oncol. 2022;29(10):6982–91. doi: 10.3390/curroncol29100549 36290826 PMC9600008

[15] Wu AD, Lindson N, Hartmann-Boyce J, Wahedi A, Hajizadeh A, Theodoulou A, et al. Smoking cessation for secondary prevention of cardiovascular disease. Cochrane Database Syst Rev. 2022;8(8):CD014936. doi: 10.1002/14651858.CD014936.pub2 35938889 PMC9358996

[16] Ikeda N, Inoue M, Iso H, Ikeda S, Satoh T, Noda M, et al. Adult mortality attributable to preventable risk factors for non-communicable diseases and injuries in Japan: A comparative risk assessment. PLoS Med. 2012;9(1):e1001160. doi: 10.1371/journal.pmed.1001160 22291576 PMC3265534

[17] Ezeamii VC, Okobi OE, Wambai-Sani H, Perera GS, Zaynieva S, Okonkwo CC, et al. Revolutionizing healthcare: How telemedicine is improving patient outcomes and expanding access to care. Cureus. 2024;16(7):e63881. doi: 10.7759/cureus.63881 39099901 PMC11298029

[18] Shi B, Li G, Wu S, Ge H, Zhang X, Chen S, et al. Assessing the effectiveness of ehealth interventions to manage multiple lifestyle risk behaviors among older adults: Systematic review and meta-analysis. J Med Internet Res. 2024;26:e58174. doi: 10.2196/58174 39083787 PMC11325121

[19] Fang YE, Zhang Z, Wang R, Yang B, Chen C, Nisa C, et al. Effectiveness of eHealth smoking cessation interventions: Systematic review and meta-analysis. J Med Internet Res. 2023;25:e45111. doi: 10.2196/45111 37505802 PMC10422176

[20] Richter KP, Shireman TI, Ellerbeck EF, Cupertino AP, Catley D, Cox LS, et al. Comparative and cost effectiveness of telemedicine versus telephone counseling for smoking cessation. J Med Internet Res. 2015;17(5):e113. doi: 10.2196/jmir.3975 25956257 PMC4468596

[21] Metin M, Kaya Ş, Sözmen K, Altınışık G. Smoking cessation support via video counseling (e-Cessation): A promising field for telemedicine implementation. Thorac Res Pract. 2024;25(3):121–9. doi: 10.5152/ThoracResPract.2024.23056 39128028 PMC11181302

[22] Nomura A, Ikeda T, Fujimoto T, Morita Y, Taniguchi C, Ishizawa T, et al. Outcomes of a telemedicine smoking cessation programme for heated tobacco product users in Japan: A retrospective cohort study. BMJ Open. 2022;12(12):e063489. doi: 10.1136/bmjopen-2022-063489 36600419 PMC9772628

[23] The data from Ministry of Health, Labour and Welfare MHLW in Japan. 2017.

[24] Kawakami N, Takatsuka N, Inaba S, Shimizu H. Development of a screening questionnaire for tobacco/nicotine dependence according to ICD-10, DSM-III-R, and DSM-IV. Addict Behav. 1999;24(2):155–66. doi: 10.1016/s0306-4603(98)00127-0 10336098

[25] Bosanquet K, Bailey D, Gilbody S, Harden M, Manea L, Nutbrown S, et al. Diagnostic accuracy of the Whooley questions for the identification of depression: A diagnostic meta-analysis. BMJ Open. 2015;5(12):e008913. doi: 10.1136/bmjopen-2015-008913 26656018 PMC4679987

[26] Nomura A, Tanigawa T, Muto T, Oga T, Fukushima Y, Kiyosue A, et al. Clinical efficacy of telemedicine compared to face-to-face clinic visits for smoking cessation: Multicenter open-label randomized controlled noninferiority trial. J Med Internet Res. 2019;21(4):e13520. doi: 10.2196/13520 30982776 PMC6660118

[27] Lee SH, Yi YH, Lee YI, Lee HY, Lim K-M. Factors associated with long-term smoking relapse in those who succeeded in smoking cessation using regional smoking cessation programs. Medicine (Baltimore). 2022;101(31):e29595. doi: 10.1097/MD.0000000000029595 35945709 PMC9351863

[28] Onda M, Horiguchi M, Domichi M, Sakane N. Effect of a smoking cessation education program on the knowledge, attitude, and self-efficacy of community pharmacists in Japan: A quasi-experimental study. Tob Use Insights. 2024;17:1179173X241272362. doi: 10.1177/1179173X241272362 39131666 PMC11311180

[29] Giummo R, Oliver JA, McClernon FJ, Sweitzer MM. Associations between compliance with very low nicotine content (VLNC) cigarettes, abstinence self-efficacy, and quit outcomes in a pilot smoking cessation trial. Drug Alcohol Depend. 2024;262:111393. doi: 10.1016/j.drugalcdep.2024.111393 39024797 PMC11360067

[30] Nur-Hasanah R, Siti Munira Y, Nadzimah MN, Mohamad Rodi I. The perceived benefits and self-efficacy of an exercise intervention on tobacco withdrawal symptoms: A qualitative study based on the health belief model. Malays J Med Sci. 2024;31(3):194–203. doi: 10.21315/mjms2024.31.3.15 38984236 PMC11229566

[31] Huo X, Li X, Gu M, Qin T, Qiao K, Bai X, et al. Mechanism of community quitters’ psychological traits on their smoking cessation effects: Based on a study of community intervention. Tob Induc Dis. 2023;21:70. doi: 10.18332/tid/162000 37252032 PMC10210092

[32] Gallus S, Cresci C, Rigamonti V, Lugo A, Bagnardi V, Fanucchi T, et al. Self-efficacy in predicting smoking cessation: A prospective study in Italy. Tob Prev Cessat. 2023;9:15. doi: 10.18332/tpc/162942 37125003 PMC10141785

[33] Barbeau EM, Krieger N, Soobader M-J. Working class matters: Socioeconomic disadvantage, race/ethnicity, gender, and smoking in NHIS 2000. Am J Public Health. 2004;94(2):269–78. doi: 10.2105/ajph.94.2.269 14759942 PMC1448243

[34] Messer K, Trinidad DR, Al-Delaimy WK, Pierce JP. Smoking cessation rates in the United States: A comparison of young adult and older smokers. Am J Public Health. 2008;98(2):317–22. doi: 10.2105/AJPH.2007.112060 18172143 PMC2376894

[35] Coambs RB, Li S, Kozlowski LT. Age interacts with heaviness of smoking in predicting success in cessation of smoking. Am J Epidemiol. 1992;135(3):240–6. doi: 10.1093/oxfordjournals.aje.a116277 1546699

[36] Guo K, Zhou L, Shang X, Yang C, E F, Wang Y, et al. Varenicline and related interventions on smoking cessation: A systematic review and network meta-analysis. Drug Alcohol Depend. 2022;241:109672. doi: 10.1016/j.drugalcdep.2022.109672 36332593

[37] Thomas KH, Dalili MN, López-López JA, Keeney E, Phillippo DM, Munafò MR, et al. Comparative clinical effectiveness and safety of tobacco cessation pharmacotherapies and electronic cigarettes: A systematic review and network meta-analysis of randomized controlled trials. Addiction. 2022;117(4):861–76. doi: 10.1111/add.15675 34636108 PMC9293179

[38] Lang AE, Berlin I. Unavailability of varenicline: A global tragedy for the fight against the tobacco epidemic. Lancet Respir Med. 2023;11(6):518–9. doi: 10.1016/S2213-2600(23)00184-4 37187193

[39] Tomioka H, Wada T, Yamazoe M, Yoshizumi Y, Nishio C, Ishimoto G. Ten-year experience of smoking cessation in a single center in Japan. Respir Investig. 2019;57(4):380–7. doi: 10.1016/j.resinv.2019.01.007 30795920

[40] Iwaoka M, Tsuji T. Twelve weeks of successful smoking cessation therapy with varenicline reduces spirometric lung age. Intern Med. 2016;55(17):2387–92. doi: 10.2169/internalmedicine.55.6844 27580538

[41] Fagerström K, Nakamura M, Cho H-J, Tsai S-T, Wang C, Davies S, et al. Varenicline treatment for smoking cessation in Asian populations: A pooled analysis of placebo-controlled trials conducted in six Asian countries. Curr Med Res Opin. 2010;26(9):2165–73. doi: 10.1185/03007995.2010.505130 20666691

