# Supplementary material for: Enhancing the long-term abstinence rate of smoking through telemedicine: A multicenter, retrospective cohort study in Japan
Source: PLoS One. 2026 Jul 22;21(7):e0331514. doi: 10.1371/journal.pone.0331514 (PMC13390940; doi:10.1371/journal.pone.0331514)

PLOS ONE

Enhancing the long-term abstinence rate of smoking through telemedicine: A multicenter, retrospective cohort study in Japan

--Manuscript Draft--

**Manuscript Number:**	PONE-D-25-20021
**Article Type:**	Research Article
**Full Title:**	Enhancing the long-term abstinence rate of smoking through telemedicine: A multicenter, retrospective cohort study in Japan
**Short Title:**	Smoking abstinence rate through telemedicine
**Corresponding Author:**	Kazuki Takakura, M.D., Ph.D.<br>Jikei University School of Medicine: Tokyo Jikeikai Ika Daigaku<br>JAPAN
**Keywords:**	smoking cessation, telemedicine, abstinence rate, completion rate, varenicline, nicotine patch
**Abstract:**	The aim of this study is to analyze the differences between telemedicine and face-to-face examinations for smoking cessation in terms of long-term abstinence and completion rates in a large Japanese population. Although the effectiveness of various smoking cessation methods has been confirmed, the treatment outcomes of face-to-face interviews for tobacco cessation are still insufficient, because it is bother to go to a clinic regularly for most patients. In line with current trends of developing digital healthcare services, we assessed the role of telemedicine as a potential tool in smoking cessation clinics. A total of 5005 current smokers who applied for the telemedicine smoking cessation program between April 2021 and December 2022 were initially included in the study. Clinical data, such as age, sex, body mass index, Brinkman index, were collected from all the participants, in addition to a preliminary questionnaire including smoking habits, reason for applying, past medical histories and self-efficacy for smoking cessation. This fully remote online program consists of four sessions in the first 8 weeks, with a 1-year follow-up period (final follow-up at 52 weeks). The success rates of long-term smoking abstinence at week 52 and the completion rates of scheduled treatment with varenicline or nicotine patches were compared between the telemedicine and face-to-face examinations. Data on face-to-face examinations were obtained from the most recent database of the Ministry of Health, Labor and Welfare in Japan for comparison. A total of 3708 patients (median age 43.6 years, 3364 men and 352 women) were analyzed. The completion rate of this program (68.2%) was significantly greater than that of face-to-face examinations (29.8%). The long-term abstinence rate was significantly greater with telemedicine than with conventional examination (P=0.000). In conclusion, the findings revealed that telemedicine is helpful for long-term abstinence from smoking on the basis of improved availability and accessibility.
**Order of Authors:**	Kazuki Takakura
	Tetsuro Ishizawa
	Yoshihiko Aoki
	Kei Matsumoto
	Toshikazu Tanaka
	Muneyuki Koyama
	Yuji Kinoshita
	Saburo Ito
	Kazuhiro Yoshiuchi
	Machi Suka
**Additional Information:**	

Powered by Editorial Manager® and ProduXion Manager® from Aries Systems Corporation

Question	Response
**Financial Disclosure**<br><br>Enter a financial disclosure statement that describes the sources of funding for the work included in this submission. Review the submission guidelines for detailed requirements. View published research articles from *PLOS ONE* for specific examples.<br><br>This statement is required for submission and **will appear in the published article** if the submission is accepted. Please make sure it is accurate.<br><br>**Funded studies**<br>Enter a statement with the following details:<br>• Initials of the authors who received each award<br>• Grant numbers awarded to each author<br>• The full name of each funder<br>• URL of each funder website<br>• Did the sponsors or funders play any role in the study design, data collection and analysis, decision to publish, or preparation of the manuscript?<br><br>Did you receive funding for this work?	The author(s) received no specific funding for this work.
**Competing Interests**<br><br>Use the instructions below to enter a competing interest statement for this submission. On behalf of all authors, disclose any competing interests that could be perceived to bias this work—acknowledging all financial support and any other relevant financial or non-financial competing interests.<br><br>This statement is required for submission and **will appear in the published article** if the submission is accepted. Please make sure it is accurate and that any funding sources listed in your Funding Information later in the submission form are also	The authors have declared that no competing interests exist.

declared in your Financial Disclosure statement.<br><br>View published research articles from *PLOS ONE* for specific examples.<br><br>**NO authors have competing interests**<br><br>Enter: *The authors have declared that no competing interests exist.*<br><br>**Authors with competing interests**<br><br>Enter competing interest details beginning with this statement:<br><br>*I have read the journal's policy and the authors of this manuscript have the following competing interests: [insert competing interests here]*<br><br>* typeset	
**Ethics Statement**<br><br>Enter an ethics statement for this submission. This statement is required if the study involved:<br><br>• Human participants<br>• Human specimens or tissue<br>• Vertebrate animals or cephalopods<br>• Vertebrate embryos or tissues<br>• Field research<br><br>Write "N/A" if the submission does not require an ethics statement.<br><br>General guidance is provided below. Consult the submission guidelines for detailed instructions. **Make sure that all information entered here is included in the Methods section of the manuscript.**	All information entered here is included in the Methods section of the manuscript.

Powered by Editorial Manager® and ProduXion Manager® from Aries Systems Corporation

**Format for specific study types**<br><br>**Human Subject Research (involving human participants and/or tissue)**<br>• Give the name of the institutional review board or ethics committee that approved the study<br>• Include the approval number and/or a statement indicating approval of this research<br>• Indicate the form of consent obtained (written/oral) or the reason that consent was not obtained (e.g. the data were analyzed anonymously)<br><br>**Animal Research (involving vertebrate animals, embryos or tissues)**<br>• Provide the name of the Institutional Animal Care and Use Committee (IACUC) or other relevant ethics board that reviewed the study protocol, and indicate whether they approved this research or granted a formal waiver of ethical approval<br>• Include an approval number if one was obtained<br>• If the study involved *non-human primates*, add *additional details* about animal welfare and steps taken to ameliorate suffering<br>• If anesthesia, euthanasia, or any kind of animal sacrifice is part of the study, include briefly which substances and/or methods were applied<br><br>**Field Research**<br><br>Include the following details if this study involves the collection of plant, animal, or other materials from a natural setting:<br>• Field permit number<br>• Name of the institution or relevant body that granted permission	
**Data Availability**<br><br>Authors are required to make all data underlying the findings described fully available, without restriction, and from the time of publication. PLOS allows rare exceptions to address legal and ethical concerns. See the PLOS Data Policy and FAQ for detailed information.	Yes - all data are fully available without restriction

Powered by Editorial Manager® and ProduXion Manager® from Aries Systems Corporation

A Data Availability Statement describing where the data can be found is required at submission. Your answers to this question constitute the Data Availability Statement and **will be published in the article**, if accepted.<br><br>**Important:** Stating 'data available on request from the author' is not sufficient. If your data are only available upon request, select 'No' for the first question and explain your exceptional situation in the text box.<br><br>Do the authors confirm that all data underlying the findings described in their manuscript are fully available without restriction?	
**Describe where the data may be found in full sentences. If you are copying our sample text, replace any instances of XXX with the appropriate details.**<br><br>• If the data are **held or will be held in a public repository**, include URLs, accession numbers or DOIs. If this information will only be available after acceptance, indicate this by ticking the box below. For example: *All XXX files are available from the XXX database (accession number(s) XXX, XXX.).*<br>• If the data are all contained **within the manuscript and/or Supporting Information files**, enter the following: *All relevant data are within the manuscript and its Supporting Information files.*<br>• If neither of these applies but you are able to provide **details of access elsewhere**, with or without limitations, please do so. For example:<br><br>*Data cannot be shared publicly because of [XXX]. Data are available from the XXX Institutional Data Access / Ethics Committee (contact via XXX) for researchers who meet the criteria for access to confidential data.*<br><br>*The data underlying the results presented in the study are available from (include the name of the third party*	N/A

and contact information or URL).<br>• This text is appropriate if the data are owned by a third party and authors do not have permission to share the data.<br><br>* typeset	
Additional data availability information:	

Manuscript
Click here to access/download;Manuscript;Manuscript for PLOS One .docx

Enhancing the long-term abstinence rate of smoking through telemedicine: A multicenter, retrospective cohort study in Japan

Kazuki Takakura[1,2]*, Tetsuro Ishizawa[3], Yoshihiko Aoki[4], Kei Matsumoto[5], Toshikazu D. Tanaka[6], Muneyuki Koyama[7], Yuji Kinoshita[8], Saburo Ito[9], Kazuhiro Yoshiuchi[3], Machi Suka[2]

[1]Department of Internal Medicine, UnMed Clinic Motomachi, Yokohama, Kanagawa, Japan.
[2]Department of Public Health and Environmental Medicine, The Jikei University School of Medicine, Tokyo, Japan.
[3]Department of Stress Sciences and Psychosomatic Medicine, Graduate School of Medicine, The University of Tokyo, Tokyo, Japan.
[4]Department of Gastroenterology and Hepatology, National Center for Global Health and Medicine Kohnodai Hospital, Chiba, Japan.
[5]Division of Nephrology and Hypertension, Department of Internal Medicine, The Jikei University School of Medicine, Tokyo, Japan.
[6]Division of Cardiology, Department of Internal Medicine, The Jikei University School of Medicine, Tokyo, Japan.
[7]Department of Surgery, The Jikei University School of Medicine, Tokyo, Japan.
[8]Division of Gastroenterology and Hepatology, Department of Internal Medicine, The Jikei University School of Medicine, Tokyo, Japan.
[9]Division of Respiratory Diseases, Department of Internal Medicine, The Jikei University School of Medicine, Tokyo, Japan.

Short Title: Smoking abstinence rate through telemedicine

Abstract

The aim of this study is to analyze the differences between telemedicine and face-to-face examinations for smoking cessation in terms of long-term abstinence and completion rates in a large Japanese population. Although the effectiveness of various smoking cessation methods has been confirmed, the treatment outcomes of face-to-face interviews for tobacco cessation are still insufficient, because it is bother to go to a clinic regularly for most patients. In line with current trends of developing digital healthcare services, we assessed the role of telemedicine as a potential tool in smoking cessation clinics. A total of 5005 current smokers who applied for the telemedicine smoking cessation program between April 2021 and December 2022 were initially included in the study. Clinical data, such as age, sex, body mass index, Brinkman index, were collected from all the participants, in addition to a preliminary questionnaire including smoking habits, reason for applying, past medical histories and self-efficacy for smoking cessation. This fully remote online program consists of four sessions in the first 8 weeks, with a 1-year follow-up period (final follow-up at 52 weeks). The success rates of long-term smoking abstinence at week 52 and the completion rates of scheduled treatment with varenicline or nicotine patches were compared between the telemedicine and face-to-face examinations. Data on face-to-face examinations were obtained from the most recent database of the Ministry of Health, Labor and Welfare in Japan for comparison. A total of 3708 patients (median age 43.6 years, 3364 men and

352 women) were analyzed. The completion rate of this program (68.2%) was significantly greater than that of face-to-face examinations (29.8%). The long-term abstinence rate was significantly greater with telemedicine than with conventional examination (P=0.000). In conclusion, the findings revealed that telemedicine is helpful for long-term abstinence from smoking on the basis of improved availability and accessibility.

Keywords: smoking cessation, telemedicine, abstinence rate, completion rate, varenicline, nicotine patch

Introduction

Although the prevalence of tobacco consumption has been gradually decreasing worldwide, the Japanese smoking rate was still 20.1% in 2020, which was almost the same as the earlier rate (1). Besides, the economic and social disparity in smoking has been expanding in Japan, similar to other developed countries, especially among the working generation (2). In addition, the success rate of continuous smoking cessation is unsatisfactory because of the limited treatment persistence rate with conventional, face-to-face examination for smoking cessation (3, 4). In current situation of overcrowded medical facilities in Japan, patients have to wait for a long time to be seen by the doctor. It makes patients feel difficulty in receiving the medication for quitting smoking. However, a wide variety of evidence demonstrating the physical, mental, financial and social benefits of smoking cessation have been accumulating (5-15). Considering not only the various health impairments stemming from smoking, but also the fact that tobacco is the primary cause of death in Japan (16), further promotion of smoking cessation and drastic measures for remedying smoking-related health inequalities at a younger age are urgently needed.

During the COVID-19 pandemic, the availability and accessibility of telemedicine, a digital health service, was promoted in various healthcare fields, including tobacco cessation clinics (17-19). Indeed, several studies have reported the usefulness of telemedicine for smoking

cessation, though limited number of patients or limited follow-up duration (20, 21). Since a previous study that used the same fully online smoking cessation program mainly focused on comparison of therapeutic outcomes between conventional cigarettes and heated tobacco products (HTPs) through telemedicine (22), we focused on comparing smoking cessation outcomes with telemedicine versus face-to-face examinations from a different perspective. Nicotine-dependent patients are commonly eligible for a 12-week standard smoking cessation treatment program with 5 clinic visits and an initial screening interview in which their exhaled carbon monoxide levels are checked in Japan (23). Therefore, the duration of this smoking cessation program with varenicline was 8 weeks in a relatively short span of time, whereas that of the conventional program is 12 weeks with varenicline.

Given the insufficient clinical evidence regarding the usefulness of telemedicine for smoking cessation, we evaluated long-term smoking abstinence rates at 52 weeks from starting the program, as well as the completion rate of the scheduled treatment for 8 weeks in comparison with face-to-face examinations in this multicenter study involving thousands of participants.

Materials and methods

Study design and population

A total of 5005 current smokers aged 20 years or older who applied for the telemedicine smoking cessation program through the health insurance association between 01/04/2021 and 31/12/2022 were potentially eligible for inclusion in this retrospective cohort study. Following enrollment into the cohort, the participants' clinical information were accessed on 01/06/2024. A total of 20 Japanese medical institutes were entered, and the number of patients enrolled were at minimum 3, and maximum 1265. Most patients were participated not as an individual but from each business site. In a preliminary questionnaire, participants were asked about their duration of smoking, previous smoking cessation history, number of cigarettes per day, reason for applying to the program past medical history and self-efficacy for quitting smoking. Clinical data, such as age, sex, body mass index (BMI), Brinkman index, the Tobacco Dependence Screener (TDS) score (24) and treatment methods were collected at baseline and in follow-up surveys. In addition, the individual's occupation, type of cigarette, drinking habits and complications of depressive symptoms identified by Whooley Questions for depression screening were also similarly recorded (25). The degree of self-efficacy for quitting smoking was assessed by dividing the participants into 3 groups: low (0-24%), medium (25-74%) and high (75-100%). For the evaluation of therapeutic efficacy, 1297 censored patients were finally

excluded from the analyses, as shown in Fig 1. A summary of the data collected from the study patients is presented in Table 1. The study protocol was approved by the ethics committee of UnMed Clinic Motomachi, Kanagawa, Japan (authorization number: UM24-01). The review board approved and waived the need for written informed consent from the participants because of the retrospective, noninterventional nature of this study. The study followed the recommended guidelines of the Declaration of Helsinki.

Fig 1. Diagram of this study. Reasons for screening failure and discontinuation from the study are indicated.

Table 1. Demographic and clinical characteristics of the study participants

Parameters	value
Age, mean (SD), years	43.6 (10.5)
Sex, n (%)	
Men	3356 (90.5)
Women	352 (9.5)
Body Mass Index, n (%) > 25	1276 (34.4)
Alcohol habits, n (%) over 4 days a week	1302 (35.1)
Occupation, n (%)	
White-collar worker	2201 (59.4)
Blue-collar worker	1507 (40.6)
Cigarettes type, n (%)	
Combustible cigarette	1499 (40.4)
Heated tobacco products	1582 (42.7)
Dual smoking	627 (16.9)
Electronic cigarette combined	798 (21.5)
Number of cigarettes per day, n(%)	54.4 (41.1)
1-20	3268 (88.1)
21-40	366(9.9)
40 >	74 (2.0)
Smoking years, n (%)	
1-20	1536 (41.4)
21-40	1916 (51.7)
40 >	256 (6.9)
Brinkman index, n(%)	
1-399	1729 (46.6)
400-699	1376 (37.1)
700 >	603 (16.3)
Tobacco Dependence Screener score, n(%)	
5-6	1046 (28.2)
7-8	1720 (46.4)
9-10	942 (25.4)
Past quit attempt, n(%)	
yes	2455 (66.2)
no	1253 (33.8)
Secondhand smoking, n (%)	1536 (41.4)
Self-efficacy, n(%)	
0-24	796 (21.5)
25-49	502 (13.5)
50-74	1821 (49.1)
75 >	589 (15.9)

A summary of qualitative and quantitative variables in the study

The telemedicine smoking cessation program

The smoking cessation program, provided online by Linkage, Tokyo, Japan, was implemented at all the clinics that participated in this study (Fig 2) (22). This fully remote online program consists of four sessions in the first 8 weeks, with a total follow-up period of 1 year via surveys and support via an app at weeks 12, 24, 36 and 52. In the telemedicine sessions, the doctors assessed the participants' progress in tobacco cessation and provided practical advice on quitting smoking each time. Varenicline or nicotine patches were selectively prescribed as anti-smoking drugs on the basis of the patient's condition. The participants could receive varenicline or nicotine patches directly at home for a total duration of 8 weeks without the need for clinic visits. Moreover, qualified experts of smoking cessation provided constant supports for the patients via email within the follow-up period.

Conventional smoking cessation program

Conventional program consists of five face-to-face examinations in the first 12 weeks, with a total follow-up period of 52 weeks. The patients selectively received varenicline for a total duration of 12 weeks or nicotine patches for a total duration of 8 weeks at each medical institutes. Data on conventional therapy were obtained from the most recent database of the Ministry of Health, Labor and Welfare (MHLW) in Japan for comparison of this study. A total of

1308 current smokers who visited some smoking cessation clinics between April 2016 and March 2017 were included in the database reported in 2017 (23). A total treatment duration with varenicline (12 weeks) is 4 weeks longer than that with the telemedicine program (8 weeks), although a total treatment duration with nicotine patches (8 weeks) and total follow-up period of 52 weeks are the same.

Fig 2. Overview of the Linkage telemedicine smoking cessation program and the image of telemedicine app.

Outcomes

The primary outcome was the long-term smoking abstinence rate at week 52 after 8 weeks of treatment with varenicline or nicotine patches following treatment through telemedicine or face-to-face interviews and its related factors.

The secondary outcome was completion rates of the scheduled treatment in the context of telemedicine versus face-to-face interviews and its related factors. Additionally, we compared the therapeutic efficacy of varenicline versus nicotine patches, a form of nicotine replacement therapy (NRT), administered through the online program.

Statistical analysis

All the statistical analyses were performed using SAS V.9.4 software (SAS Institute, Cary, NC, USA). The percentages of patients who completed the scheduled treatment with varenicline and nicotine patches were compared using the χ-square test. Completion rates were compared with those reported by the MHLW using the one-sample Z-test. Multiple logistic regression analysis was performed to identify factors associated with treatment completion. Odds ratios (ORs) and 95% confidence intervals (CIs) for treatment completion were calculated using multivariate adjustment. The significance level was set at $p<0.05$.

Results

Participant characteristics

A total of 3708 patients who participated in the smoking cessation program at several medical institutes between April 2021 and December 2022 were included in the analyses (Figure 1). The baseline characteristics of the study participants are summarized in Table 1. The median age of the enrolled patients was 43.6±10.5 years, with 3364 (90.5%) men and 352 (9.5%) women. Among them, 65.5% were underweight or of normal weight (BMI < 25 kg/m^2), and 34.4% were overweight (BMI ≥ 25 kg/m^2). The types of smoking products included combustible cigarettes in 40.4%, and HTP in 42.7%, while 16.9% were dual users. Although the number of cigarettes smoked was < 20 per day in 88.1% of the participants, the duration of smoking ranged from 1-20 years in 41.5%, 21-40 years in 51.6% and more than 40 years in 6.9%. Alcohol was consumed by 1694 participants (45.6%), and 987 (26.6%) had mental illnesses. In terms of occupation, blue- and white-collar workers numbered 1514 (40.7%) and 2202 (59.3%), respectively. According to the preliminary questionnaire, the degree of self-efficacy at the initiation of treatment was low in 21.5%, medium in 62.6% and high in 15.9% of the study participants.

High completion rate of scheduled treatment through telemedicine

The completion rate of scheduled treatment in the fully online program (68.2%) was significantly higher than that of face-to-face examinations (34.6%) reported by the MHLW. This was possibly due to the easy accessibility of telemedicine without the need for clinic visits and the availability of direct home-delivery of the drugs. Moreover, varenicline (78.8%) showed significantly higher completion rates than nicotine patches (65.4%) in the online program, similar to conventional treatment (P<0.001) (Table 2).

Table 2. The completion and abstinence rate of scheduled treatment for 8 weeks

	n	Completion (n, %)		Abstinence (n, %)	
Total	3708	3411	92.0	2529	68.2
varenicline	805	747	92.8	634	78.8
nicotine patch	2893	2659	91.9	1893	65.4
None	10	5	50.00	2	20.0
			**P = 0.411**		**P < 0.001**

Factors related to the completion rate according to logistic regression analysis

As shown in Table 3, older age (OR 1.99; 95% CI 1.40-2.82), HTP user (OR 1.37; 95% CI 1.02-1.84), a higher Brinkmann index (OR 1.07; 95% CI 1.00-1.15) and higher self-efficacy (OR 1.08; 95% CI 1.03-1.14) were significant factors in the completion rate of the scheduled treatment according to logistic regression analysis. Conversely, sex, BMI, alcohol habits, occupation, mental disorders, TDS score and non-smoker status were not significantly correlated with the completion rate of the scheduled treatment.

Table 3. Related factors for the completion rate of scheduled treatment

Parameters	total	completion	%	p	OR	95%CI
Age						
20-44	1840	1636	88.9	**<0.001**	1	
45 <	1858	1770	95.3		**1.99**	1.40-2.82
Sex						
Men	3347	3083	92.1	0.953	1	
Women	351	323	92.0		0.98	0.65-1.49
Body Mass Index						
> 25	2427	2237	92.2	0.833	1	
< 25	1271	1169	92.0		0.89	0.69-1.15
Alcohol habits						
within 0-3 days a week	2400	2203	91.8	0.339	1	
over 4 days a week	1298	1203	92.7		0.92	0.70-1.20
Occupation						
White-collar worker	2194	2042	93.1	0.008	1	
Blue-collar worker	1504	1364	90.7		0.84	0.66-1.07
Depressive symptoms						
none	2718	2509	92.3	0.438	1	
yes	980	897	91.5		1.02	0.77-1.34
Cigarettes type						
Combustible cigarette	1495	1378	92.2	**<0.001**	1	
Heated tobacco products	1578	1478	93.7		**1.37**	1.02-1.84
Dual smoking	625	550	88		**0.72**	0.52-0.99
Electronic cigarette combined	795	734	92.3		1.05	0.77-1.44
Brinkman index						
1-399	1722	1542	89.5	**<0.001**	**1.07**	1.00-1.15
400-699	1375	1298	94.4			
700 >	601	566	94.2			
Tobacco Dependence Screener score						
5-6	1040	972	93.5	0.076	1	0.92-1.10
7-8	1719	1566	91.1			
9-10	939	869	92.4			
Past quit attempt						
none	1285	1177	91.6	0.403	1	
yes	2413	2229	92.4		1.02	0.79-1.31
Self-efficacy						
0-24	795	710	89.3	**0.003**	**1.08**	1.03-1.14
25-49	502	457	91.0			
50-74	1818	1694	93.2			
75 >	583	545	93.5			
Drug						
varenicline	805	747	92.8	0.411	1	
nicotine patch	2893	2659	91.9		0.95	0.70-1.29

Telemedicine enhanced long-term smoking cessation rates

A total of 3698 patients were included in the comparative analysis of the results of the 1-year follow-up. Four hundred thirty-three of 805 varenicline users (53.8%) and 1230 out of 2893 nicotine patch users (42.5%) were able to quit smoking continuously, even though only 28.3% of the varenicline users and 34.5% of the nicotine patch users were in the same setting reported from the MHLW in Japan (Table 4). Taken together, the long-term abstinence rate was significantly greater with telemedicine rather with conventional examination regardless of the therapeutic method used (P 0.000).

Table 4. The comparison of the long-term abstinence rate at 52 weeks

**Full online program**	total	abstinence	%
varenicline	805	433	53.8
nocotine patch	2893	1230	42.5
**Face-to-face examination (data from MHLW)**			
varenicline	916	259	28.3
nicotine patch	145	50	34.5
**the difference in the population proportions**	Z	p	
varenicline	16.015	**< 0.0001**	
nicotine patch	9.051	**< 0.0001**	

MHLW: Ministry of Health, Labour and Welfare

Factors related to the abstinence rate according to logistic regression analysis

Next, we assessed factors related to the abstinence rate at the 1-year follow-up from therapeutic initiation. As shown in Table 5, older age (OR 1.60; 95% CI 1.34-1.91), blue-collar workers (OR 0.85; 95% CI 0.74-0.98), depressive symptoms (OR 0.82; 95% CI 0.71-0.96), HTP users (OR 1.34; 95% CI 1.15-1.56) and higher self-efficacy (OR 1.05; 95% CI 1.02-1.08) were significantly related to outcomes. In addition, participants who used varenicline had a higher abstinence rate than those who received nicotine patches in the online program (OR 0.66; 95% CI 0.57-0.78). On the other hand, sex, BMI, alcohol habit, Brinkman index, TDs score and non-smoking status were not correlated with the long-term abstinence rate.

Table 5. Related factors for the long-term abstinence rate at 52 weeks

Parameters	OR	95%CI
**Age**		
20-44	1	
45 <	**1.6**	1.34-1.91
**Sex**		
Men	1	
Women	0.94	0.83-1.16
**Body Mass Index**		
< 25	1	
> 25	1.04	0.91-1.20
**Alcohol habits**		
within 0-3 days a week	1	
over 4 days a week	1.03	0.90-1.19
**Occupation**		
White-collar worker	1	
Blue-collar worker	**0.85**	0.74-0.98
**Depressive symptoms**		
none	1	
yes	**0.82**	0.71-0.96
**Cigarettes type**		
Combustible cigarette	1	
Heated tobacco products	**1.34**	1.15-1.56
Dual smoking	**0.73**	0.60-0.90
Electronic cigarette combined	0.93	0.78-1.10
**Brinkman index**	1.02	0.99-1.05
**Tobacco Dependence Screener score**	0.98	0.93-1.02
**Past quit attempt**		
none	1	
yes	0.99	0.87-1.15
**Self-efficacy**		
0-24	**1.05**	1.02-1.08
25-49		
50-74		
75 >		
**Drug**		
varenicline	1	
nicotine patch	**0.66**	0.57-0.78

Discussion

This multicenter, retrospective cohort study revealed significant difference in long-term smoking abstinence rates between the group treated through telemedicine and the group treated through face-to-face interviews at 52 weeks from treatment initiation. The improvement in the abstinence rates observed in the present study highlights the better completion rate of the scheduled treatment with the online program, likely since it is a more convenient therapeutic method and contributes to enhance self-efficacy for quitting smoking. Telemedicine allows smokers to undergo medical consultations and to receive prescriptions without the need for clinic visits, whereas a total of 5 times follow-up visits is needed in face-to-face examinations. Moreover, it is conceivable that the constant supports from our experts of smoking cessation within the 1-year follow-up also contributed for this online program. A previous report from Japan, which also compared the results of telemedicine versus conventional examinations, evaluated short-term abstinence rates for smoking cessation (26). Another study from the United States assessed telemedicine versus telephone counseling for smoking abstinence at month 12 (20). In addition, a report from Turkey showed the utility of telemedicine in comparison to traditional counseling by evaluating smoking cessation status at 6-9 months of follow-up (21). To the best of our knowledge, this is the first large-scale study to compare the long-term smoking abstinence rates achieved by telemedicine versus face-to-face examination. Since the

risk of smoking relapse is particularly high within the first year after cessation, we followed the patients for 1 year after treatment initiation in this study and evaluated the therapeutic outcomes at that time (27). Thus, our results help elucidate the potential role of telemedicine in smoking cessation.

In this study, it is noteworthy that self-efficacy was a significant factor not only for the completion rate, but also for the abstinence rate. The results suggest that a telemedicine-supported program has a greater impact on improving self-efficacy for tobacco cessation, leading to better outcomes. The fact that self-efficacy is closely related to supporting smoking cessation is already well known (28-32). Our data are consistent with previous studies and suggest that self-efficacy in quitting smoking can be positively influenced by the availability of treatment.

Interestingly, occupation, whether the participant was a blue-collar or white-collar worker, was a significant factor in the long-term abstinence rate, although there was no correlation with age. As previously reported, responding to socioeconomic inequalities in relation to smoking cessation still remains a task for the future (2, 33). Additionally, since several studies have indicated that younger smokers are more likely to be able to quit smoking successfully than older smokers are, further investigations into age-related factors for successful smoking cessation are needed (34, 35).

In the comparison of varenicline and NRT in the online program, varenicline yielded better results than nicotine patch in terms of both the completion rate and the long-term abstinence rate, similar to the results for face-to-face examinations (36, 37). Although, varenicline is currently not readily available in Japan, the resumption of varenicline sales is expected very quickly (38).

This study has some limitations. First, the participants in this program were all members of the Japanese health insurance association, i.e. they were all within the working generation. Hence, it is likely that there is some selection bias in this study. Second, a total treatment duration with varenicline through this online program was 8 weeks, even though varenicline is usually prescribed for 12 weeks at conventional, face-to-face clinics (39-41). For a valid comparison, these two programs should be similar to ensure that any differences are due to the delivery format rather than other factors. Thus, we compared the study data with pre-existing data reported from the MHLW by setting the same follow-up period of 52 weeks from treatment initiation, to unify the total follow-up period.

A key strength of our study was the large number of participants included from multiple medical institutions with complete follow-up for 52 weeks. Our study provides definitive insights into continuous smoking abstinence and the potential efficacy of telemedicine in smoking cessation.

In conclusion, telemedicine is definitely helpful for long-term abstinence from smoking, on the

basis of its improved availability and accessibility. The higher completion rate of the scheduled treatment was likely a key player in the observed therapeutic outcomes. In addition, the resumption of varenicline sales is desirable, in view of its better outcomes than NRT in telemedicine, which is similar to conventional examinations.

Contributions

Conceptualization: Kazuki Takakura, Tetsuro Ishizawa, Kazuhiro Yoshiuchi, Machi Suka.

Data curation: Yoshihiko Aoki, Kei Matsumoto, Toshikazu D. Tanaka, Muneyuki Koyama, Yuji Kinoshita, Saburo Ito.

Investigation: Yoshihiko Aoki, Kei Matsumoto, Toshikazu D. Tanaka, Muneyuki Koyama, Yuji Kinoshita, Saburo Ito.

Project administration: Kazuki Takakura, Tetsuro Ishizawa.

Resources: Tetsuro Ishizawa.

Software: Machi Suka.

Supervision: Kazuhiro Yoshiuchi, Machi Suka.

Writing–original draft: Kazuki Takakura.

Writing–review & editing: Kazuki Takakura, Tetsuro Ishizawa, Machi Suka.

References

1. WHO global report on trends in prevalence of tobacco use 2000–2030. Geneva: World Health Organization; 2024.2024.
2. Tanaka H, Mackenbach JP, Kobayashi Y. Widening Socioeconomic Inequalities in Smoking in Japan, 2001-2016. J Epidemiol. 2021;31(6):369-77.
3. Hughes JR, Peters EN, Naud S. Relapse to smoking after 1 year of abstinence: a meta-analysis. Addict Behav. 2008;33(12):1516-20.
4. Agboola SA, Coleman T, McNeill A, Leonardi-Bee J. Abstinence and relapse among smokers who use varenicline in a quit attempt-a pooled analysis of randomized controlled trials. Addiction. 2015;110(7):1182-93.
5. Khalifeh M, Ginex P, Boffetta P. Reduction of head and neck cancer risk following smoking cessation: a systematic review and meta-analysis. BMJ Open. 2024;14(8):e074723.
6. Ravidà A, Saleh MHA, Ghassib IH, Qazi M, Kumar PS, Wang HL, et al. Impact of smoking on cost-effectiveness of 10-48 years of periodontal care. Periodontol 2000. 2024.
7. Ayaz D, Asi E, Meydanlioglu A, Oncel S. Effectiveness of smoking cessation interventions in the workplace: A systematic review and meta-analysis. Am J Ind Med. 2024;67(8):712-22.
8. Walicka M, Krysiński A, La Rosa GRM, Sun A, Campagna D, Di Ciaula A, et al. Influence of quitting smoking on diabetes-related complications: A scoping review with a systematic search strategy. Diabetes Metab Syndr. 2024;18(5):103044.
9. Lai H, Liu Q, Ye Q, Liang Z, Long Z, Hu Y, et al. Impact of smoking cessation duration on lung cancer mortality: A systematic review and meta-analysis. Crit Rev Oncol Hematol. 2024;196:104323.
10. Wahbeh F, Restifo D, Laws S, Pawar A, Parikh NS. Impact of tobacco smoking on disease-specific outcomes in common neurological disorders: A scoping review. J Clin Neurosci. 2024;122:10-8.
11. Higashi Y. Smoking cessation and vascular endothelial function. Hypertens Res. 2023;46(12):2670-8.
12. Halms T, Strasser M, Hasan A, Rüther T, Trepel M, Raab S, et al. Smoking and quality of life in lung cancer patients: systematic review. BMJ Support Palliat Care. 2024;13(e3):e686-e94.
13. Delcroix MH, Delcroix-Gomez C, Marquet P, Gauthier T, Thomas D, Aubard Y. Active or passive maternal smoking increases the risk of low birth weight or preterm delivery: Benefits of cessation and tobacco control policies. Tob Induc Dis. 2023;21:72.
14. Hoch JS, Barr HK, Guggenbickler AM, Dewa CS. Lessons from Cost-Effectiveness Analysis of Smoking Cessation Programs for Cancer Patients. Curr Oncol. 2022;29(10):6982-91.

15. Wu AD, Lindson N, Hartmann-Boyce J, Wahedi A, Hajizadeh A, Theodoulou A, et al. Smoking cessation for secondary prevention of cardiovascular disease. Cochrane Database Syst Rev. 2022;8(8):Cd014936.

16. Ikeda N, Inoue M, Iso H, Ikeda S, Satoh T, Noda M, et al. Adult mortality attributable to preventable risk factors for non-communicable diseases and injuries in Japan: a comparative risk assessment. PLoS Med. 2012;9(1):e1001160.

17. Ezeamii VC, Okobi OE, Wambai-Sani H, Perera GS, Zaynieva S, Okonkwo CC, et al. Revolutionizing Healthcare: How Telemedicine Is Improving Patient Outcomes and Expanding Access to Care. Cureus. 2024;16(7):e63881.

18. Shi B, Li G, Wu S, Ge H, Zhang X, Chen S, et al. Assessing the Effectiveness of eHealth Interventions to Manage Multiple Lifestyle Risk Behaviors Among Older Adults: Systematic Review and Meta-Analysis. J Med Internet Res. 2024;26:e58174.

19. Fang YE, Zhang Z, Wang R, Yang B, Chen C, Nisa C, et al. Effectiveness of eHealth Smoking Cessation Interventions: Systematic Review and Meta-Analysis. J Med Internet Res. 2023;25:e45111.

20. Richter KP, Shireman TI, Ellerbeck EF, Cupertino AP, Catley D, Cox LS, et al. Comparative and cost effectiveness of telemedicine versus telephone counseling for smoking cessation. J Med Internet Res. 2015;17(5):e113.

21. Metin M, Kaya Ş, Sözmen K, Altınışık G. Smoking Cessation Support via Video Counseling (e-Cessation): A Promising Field for Telemedicine Implementation. Thorac Res Pract. 2024;25(3):121-9.

22. Nomura A, Ikeda T, Fujimoto T, Morita Y, Taniguchi C, Ishizawa T, et al. Outcomes of a telemedicine smoking cessation programme for heated tobacco product users in Japan: a retrospective cohort study. BMJ Open. 2022;12(12):e063489.

23. The data from Ministry of Health, Labour and Welfare (MHLW) in Japan. 2017.

24. Kawakami N, Takatsuka N, Inaba S, Shimizu H. Development of a screening questionnaire for tobacco/nicotine dependence according to ICD-10, DSM-III-R, and DSM-IV. Addict Behav. 1999;24(2):155-66.

25. Whooley MA, Avins AL, Miranda J, Browner WS. Case-finding instruments for depression. Two questions are as good as many. J Gen Intern Med. 1997;12(7):439-45.

26. Nomura A, Tanigawa T, Muto T, Oga T, Fukushima Y, Kiyosue A, et al. Clinical Efficacy of Telemedicine Compared to Face-to-Face Clinic Visits for Smoking Cessation: Multicenter Open-Label Randomized Controlled Noninferiority Trial. J Med Internet Res. 2019;21(4):e13520.

27. Lee SH, Yi YH, Lee YI, Lee HY, Lim KM. Factors associated with long-term smoking relapse in those who succeeded in smoking cessation using regional smoking cessation programs. Medicine (Baltimore). 2022;101(31):e29595.

28. Onda M, Horiguchi M, Domichi M, Sakane N. Effect of a Smoking Cessation Education Program on the Knowledge, Attitude, and Self-Efficacy of Community Pharmacists in Japan: A Quasi-Experimental Study. Tob Use Insights. 2024;17:1179173x241272362.

29. Giummo R, Oliver JA, McClernon FJ, Sweitzer MM. Associations between compliance with very low nicotine content (VLNC) cigarettes, abstinence self-efficacy, and quit outcomes in a pilot smoking cessation trial. Drug Alcohol Depend. 2024;262:111393.

30. Nur-Hasanah R, Siti Munira Y, Nadzimah MN, Mohamad Rodi I. The Perceived Benefits and Self-Efficacy of an Exercise Intervention on Tobacco Withdrawal Symptoms: A Qualitative Study Based on the Health Belief Model. Malays J Med Sci. 2024;31(3):194-203.

31. Huo X, Li X, Gu M, Qin T, Qiao K, Bai X, et al. Mechanism of community quitters' psychological traits on their smoking cessation effects: Based on a study of community intervention. Tob Induc Dis. 2023;21:70.

32. Gallus S, Cresci C, Rigamonti V, Lugo A, Bagnardi V, Fanucchi T, et al. Self-efficacy in predicting smoking cessation: A prospective study in Italy. Tob Prev Cessat. 2023;9:15.

33. Barbeau EM, Krieger N, Soobader MJ. Working class matters: socioeconomic disadvantage, race/ethnicity, gender, and smoking in NHIS 2000. Am J Public Health. 2004;94(2):269-78.

34. Messer K, Trinidad DR, Al-Delaimy WK, Pierce JP. Smoking cessation rates in the United States: a comparison of young adult and older smokers. Am J Public Health. 2008;98(2):317-22.

35. Coambs RB, Li S, Kozlowski LT. Age interacts with heaviness of smoking in predicting success in cessation of smoking. Am J Epidemiol. 1992;135(3):240-6.

36. Guo K, Zhou L, Shang X, Yang C, E F, Wang Y, et al. Varenicline and related interventions on smoking cessation: A systematic review and network meta-analysis. Drug Alcohol Depend. 2022;241:109672.

37. Thomas KH, Dalili MN, López-López JA, Keeney E, Phillippo DM, Munafò MR, et al. Comparative clinical effectiveness and safety of tobacco cessation pharmacotherapies and electronic cigarettes: a systematic review and network meta-analysis of randomized controlled trials. Addiction. 2022;117(4):861-76.

38. Lang AE, Berlin I. Unavailability of varenicline: a global tragedy for the fight against the tobacco epidemic. Lancet Respir Med. 2023;11(6):518-9.

39. Tomioka H, Wada T, Yamazoe M, Yoshizumi Y, Nishio C, Ishimoto G. Ten-year experience of smoking cessation in a single center in Japan. Respir Investig. 2019;57(4):380-7.

40. Iwaoka M, Tsuji T. Twelve Weeks of Successful Smoking Cessation Therapy with Varenicline Reduces Spirometric Lung Age. Intern Med. 2016;55(17):2387-92.

41. Fagerström K, Nakamura M, Cho HJ, Tsai ST, Wang C, Davies S, et al. Varenicline

treatment for smoking cessation in Asian populations: a pooled analysis of placebo-controlled trials conducted in six Asian countries. Curr Med Res Opin. 2010;26(9):2165-73.

Acknowledgements

We would like to express our sincere gratitude to Ryosuke Onishi of Linkage, Inc, Tokyo, Japan for his cooperation in this study. The authors thank FORTE Science Communications (https://www.forte-science.co.jp/) for English language editing.

Funding

The authors received no specific funding for this work.

Data availability

Data supporting the findings of this study are available upon request from the corresponding author. The data are not publicly available due to ethical restrictions.

Ethics declarations

This study was approved by the ethical committee of UnMed Clinic Motomachi (UM24-01).

Consent for publication

Not applicable.

Competing interests

All authors declare that they have no competing interests.

Figure legends

Fig 1. Diagram of this study. Reasons for screening failure and discontinuation from the study are indicated.

Fig 2. Overview of the Linkage telemedicine smoking cessation program and the image of telemedicine app.

 Click here to access/download;Figure;Fig 1.TIF

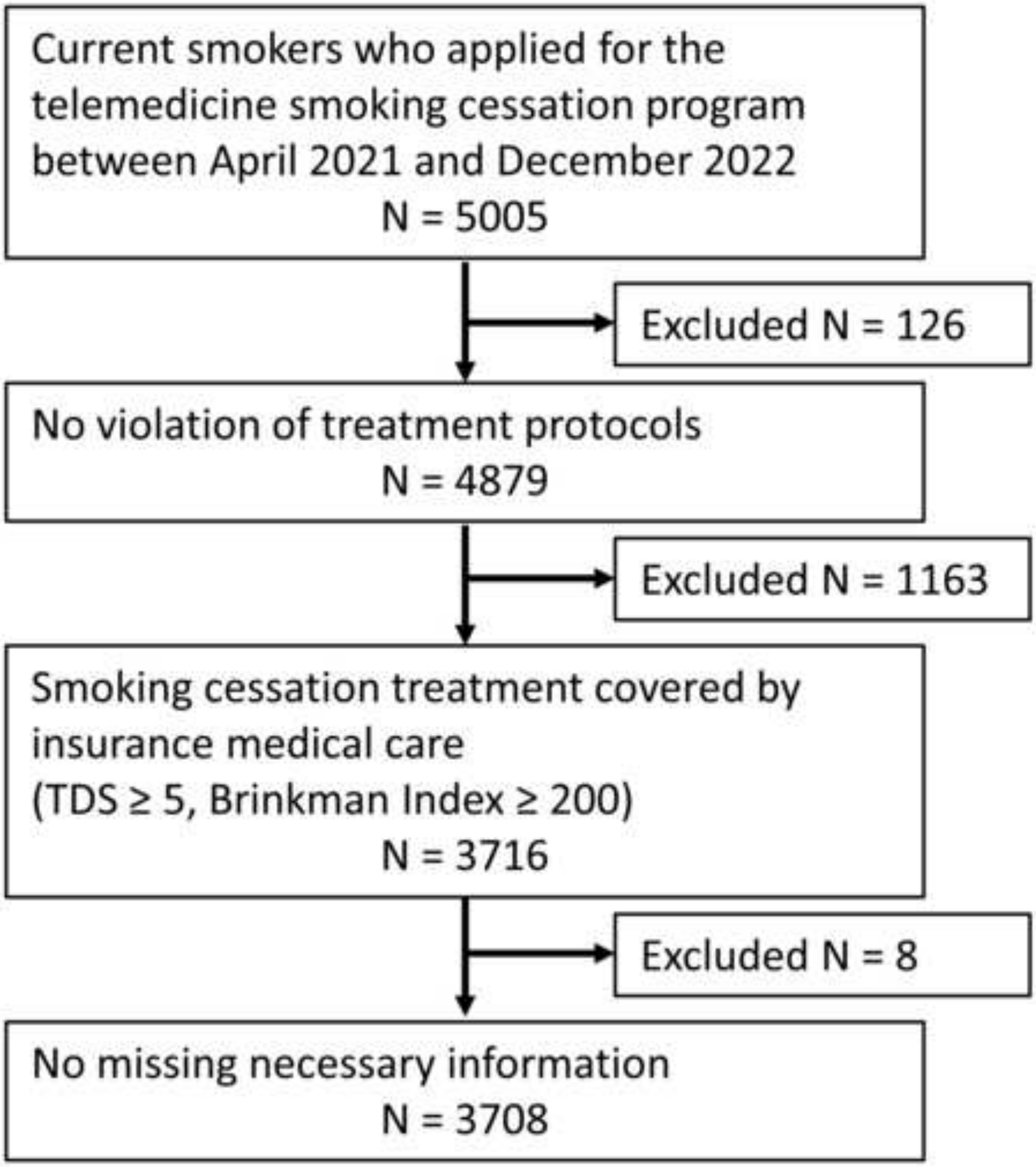

Fig 2

Click here to access/download;Figure;Fig 2.TIF

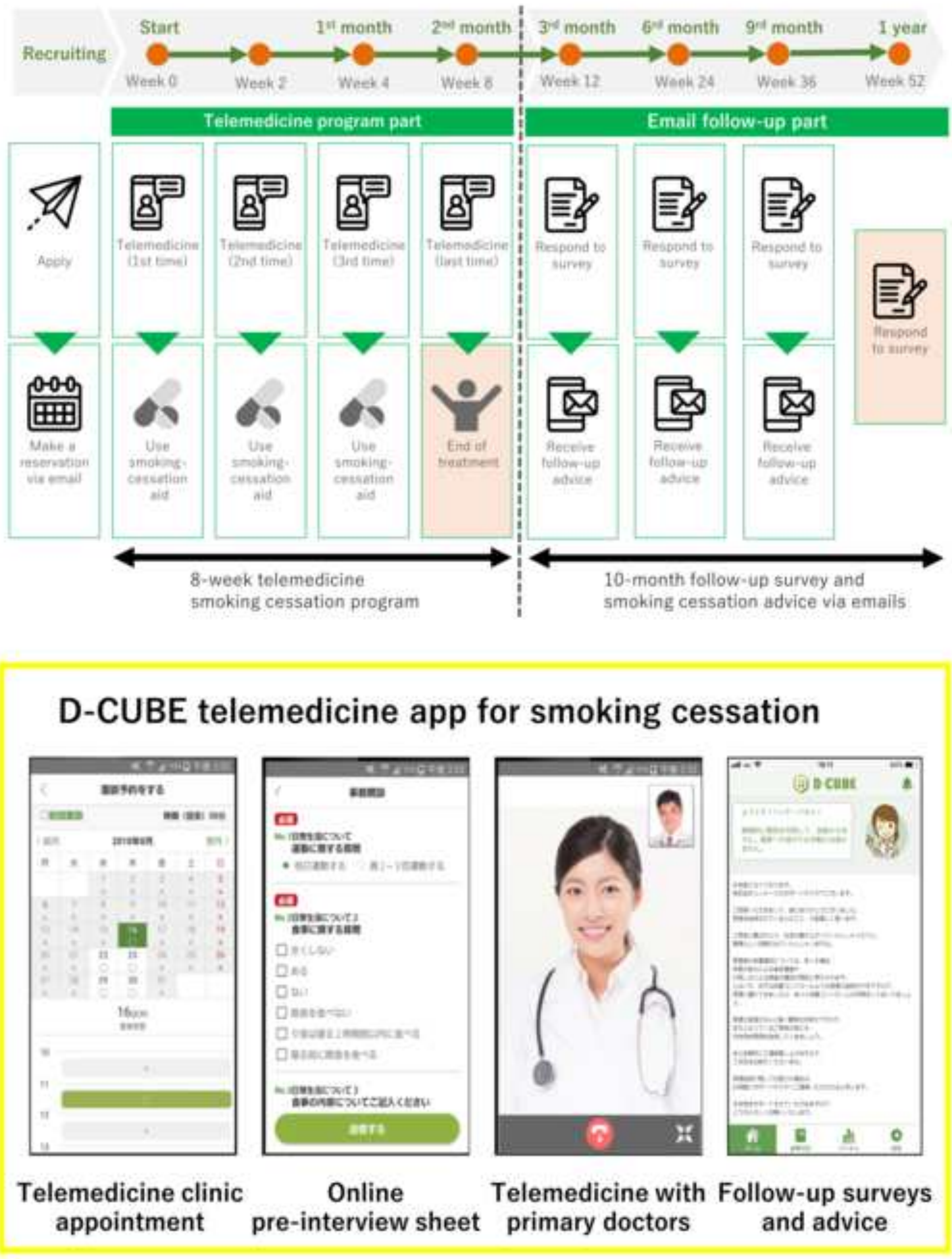

Supplement: Attachment — Submitted filename: Completed - PONE-D-25-20021_reviewer.pdf [file pone.0331514.s001.pdf]
