# Supplementary material for: Enhancing the long-term abstinence rate of smoking through telemedicine: A multicenter, retrospective cohort study in Japan
Source: PLoS One. 2026 Jul 22;21(7):e0331514. doi: 10.1371/journal.pone.0331514 (PMC13390940; doi:10.1371/journal.pone.0331514)
Supplement: Attachment — Submitted filename: Response to Reviewers.docx [file pone.0331514.s002.docx]

**Response to Reviewers**

October 26, 2025

Dear Editor,

Thank you for giving me the opportunity to submit a revised draft of my manuscript titled **“Enhancing the long-term abstinence rate of smoking through telemedicine: A multicenter, retrospective cohort study in Japan”** to *PLOS ONE*. We appreciate the time and effort that you and the reviewers have dedicated to providing your valuable feedback on my manuscript. We are grateful to the reviewers for their insightful comments on my paper. We have been able to incorporate changes to reflect most of the suggestions provided by the reviewers. Here is a point-by-point response to the reviewers’ comments and concerns.

**Comments from Journal**

When submitting your revision, we need you to address these additional requirements.

1. Please ensure that your manuscript meets PLOS ONE's style requirements, including those for file naming. The PLOS ONE style templates can be found at

<https://journals.plos.org/plosone/s/file?id=wjVg/PLOSOne_formatting_sample_main_body.pdf> and

<https://journals.plos.org/plosone/s/file?id=ba62/PLOSOne_formatting_sample_title_authors_affiliations.pdf>

→ We tried for our manuscript to meet your style requirements.

2. Your ethics statement should only appear in the Methods section of your manuscript. If your ethics statement is written in any section besides the Methods, please delete it from any other section.

→We deleted the statement from any other section.

3. Please provide a complete Data Availability Statement in the submission form, ensuring you include all necessary access information or a reason for why you are unable to make your data freely accessible. If your research concerns only data provided within your submission, please write "All data are in the manuscript and/or supporting information files" as your Data Availability Statement.

→ We provided a complete Data Availability Statement in the submission form.

4. If the reviewer comments include a recommendation to cite specific previously published works, please review and evaluate these publications to determine whether they are relevant and should be cited. There is no requirement to cite these works unless the editor has indicated otherwise.

→ We got it.

**Comments from Reviewer 1**

- Participants were self-selected individuals who applied for a telemedicine smoking cessation program via their health insurance association. This may lead to a population with higher motivation or resources for quitting smoking, which limits generalizability to all smokers.
-The cohort consisted of Japanese workers participating in a health-insurance-linked program, with most joining via their business sites. Findings may not generalize to unemployed individuals, non-Japanese populations, or those without access to similar healthcare systems or programs.
Response: Thank you for the comments.

We understood that the points raised in these two comments are largely similar. We agree with the comments. Therefore, we added the explanation as a limitation in Discussion section.

- Data were collected from 20 medical institutions with widely varying sample sizes.
Variability in patient volume and potentially in clinical practices between sites could introduce site-level confounding or bias.

Response: Thank you for pointing this out. However, the attributes of all of the participating medical institutes of this study are consistent as a clinic, not large hospital. It is not unusual that there are a lot of low-volume clinics, on the basis of a statistical result showing that average number of patients who want to quit smoking per facility per year is 12.8. In addition, a certain level of medical skill is guaranteed in the program, because all of the attending doctors basically completed the required online learning about smoking cessation.

- Data such as smoking history, self-efficacy, and depressive symptoms were based on self-reported questionnaires. This could lead to recall bias and misclassification.

-1,297 patients were excluded due to censoring (unclear follow-up status, lost to follow-up)
This could result in attrition bias.
Response: Thank you for the comments.

We agree with both of the comments. We have accordingly added the sentences as limitations in Discussion section.

**Comments from Reviewer 2**

Thank you for the opportunity to review the manuscript which addresses a clinically important topic and contributes valuable insights into the comparative effectiveness of telemedicine vs face to face intervention in smoking cessation. Smoking is a major public health problem worldwide and addressing this issues and the investigation into telemedicine-based smoking cessation aligns with global shift in healthcare delivery, specially post COVID. The inclusion of over 3700 participants across multiple sites enhances the generalizability of findings with in the Japanese healthcare context. Clear primary and secondary outcomes, comparison with data from japan's MHLW adds contextual strengths and relevance.

Response: Thank you for your positive feedback.

Authors have acknowledged potential selection bias, please clarify how patients were assigned face to face vs telemedicine on the basis of their preference, clinic assignment or another factor?

Response: Thank you for pointing this out. However, as mentioned in Materials and methods section, we just provided the online program only. And a comparison target of face-to-face was prepared from the database of the Ministry of Health, Labor and Welfare (MHLW) in Japan. Therefore, all of the included patient made decisions based on their own will.

The manuscript contains frequent grammatical errors, would recommend proof reading.

Response: Thank you for the comments.

Actually, we have used an editing service by a native English speaker for this manuscript. I attached the certificate.

In intervention, consider including more details about the content and format of the 4 telemedicine sessions. Were there any specific modality of therapy, CBT, MI was utilized
Response: Thank you for the comments.

Figure 2 shows in detail about the online program with 4 telemedicine sessions. All of the attending doctors basically completed the required online learning about smoking cessation counseling and approach the smoking cessation program with understanding the cognitive behavioral therapy. Therefore, all of the patients were treated similarly without any specific therapeutic modality in the program.

**Comments from Reviewer 3**
The abstract needs to state how many participants for face-to-face. The results should highlight both telemedicine and face-to-face.
Response: Thank you for pointing this out.

We agree with this comment. We have accordingly modified the sentences in Abstract.

Line 46: Technically, the p-value cannot be zero (to use the symbol p < )
Response: Thank you for the comments.

We modified the P value to <0.005.

Table 1: 40 > to be revised to >40. Likewise for others 700 > and 75 >4 (to use ≤ or ≥ where applicable)
Response: Thank you for the comments.

We corrected the point in Table 1.

Table 1: Face-to-face data is to be included for comparison.
Response: Thank you for the comments.

However, it is difficult to include face-to-face data in Table 1. Because these targeted patients are different with online and face-to-face. Instead, we gave a supplementary explanation about that in Results section.

Table 3: 45< , > 25, 700 >, 75 > symbol used incorrectly (to use ≤ or ≥ where applicable). Response: Thank you for the comments.

We corrected the point in Table 3.

Figure 1: The figure requires revision to show the face-to-face arm.
Response: Thank you for the comments.

However, Figure 1 just showed the diagram of the patients treated from us through online. Then, we didn’t treat for the patients of the face-to-face group. Therefore, we modified the Figure legends appropriately.

Line 161-174: The section is to be divided according to telemedicine and face-to-face.
Response: Thank you for pointing this out.

We agree with this comment. We have accordingly added explanation for face-to-face group in the section.

Table 2: Face-to-face data to be presented along with telemedicine data.
Response: Thank you for the comments.

However, it is too complicated if face-to-face data is also demonstrated in Table 2. Because the treatment duration with varenicline is 12 weeks in face-to-face examination (4 weeks longer than online examination), even though the treatment duration with nicotine patch is same.

Line 184: To indicate the p-values for the comparison between completed vs not completed and abstinence vs non-abstinence.
Response: Thank you for the comments.

Unfortunately, there is no data available for the p-values for the comparison from the Ministry of Health, Labor and Welfare in Japan.

Table 3: Separate analyses to be provided for telemedicine and face-to-face. Likewise, for Table 5.
Response: Thank you for the comments.

Unfortunately, there is no data available for the specific related factors of face-to-face examinations from the Ministry of Health, Labor and Welfare in Japan.

Line 202: (P 0.000)?
Response: Thank you for the comments.

We modified the P value to <0.001.

Table 5: 45 <, > 25, over 4 days a week, 75 >, incorrect symbol was used. To use ≤ or ≥ where applicable. P-value is to be presented.
Response: Thank you for the comments.

We corrected the point in Table 5 added the p-value.

Please remove the underline for the confidence interval value, since the p-value is sufficient to indicate statistical significance. Similarly, the odds ratios (OR) should not be in bold.
For the adjustment analyses, the words adjusted and aOR are to be used where appropriate, including tables.
The fulfilment of the statistical tests' assumptions is to be stated e.g multicollinearity etc.
Model fit etc for logistic regression is to be presented.

Response: Thank you for the comments.

We corrected the points in all of the Tables.

In addition, the fulfilment of the statistical test’s assumptions is stated in “Statistical analysis” section.

Sincerely,

Kazuki Takakura, M.D., Ph. D.

UnMed Clinic Motomachi

3-116 Motomachi, Naka-ku, Yokohama, Kanagawa 231-0861, Japan

E-mail: ktakakura@jikei.ac.jp
