# Supplementary material for: Enhancing the long-term abstinence rate of smoking through telemedicine: A multicenter, retrospective cohort study in Japan
Source: PLoS One. 2026 Jul 22;21(7):e0331514. doi: 10.1371/journal.pone.0331514 (PMC13390940; doi:10.1371/journal.pone.0331514)
Supplement: Attachment — Submitted filename: Response to Reviewers.(3) docx.docx [file pone.0331514.s004.docx]

**Response to Reviewers**

March 17, 2026

Dear Editor,

Thank you for giving me the opportunity to submit a revised draft of my manuscript titled **“Enhancing the long-term abstinence rate of smoking through telemedicine: A multicenter, retrospective cohort study in Japan”** to *PLOS ONE*. We appreciate the time and effort that you and the reviewers have dedicated to providing your feedback on my manuscript.

Reviewer's Responses to Questions

**Comments to the Author**

1. If the authors have adequately addressed your comments raised in a previous round of review and you feel that this manuscript is now acceptable for publication, you may indicate that here to bypass the “Comments to the Author” section, enter your conflict of interest statement in the “Confidential to Editor” section, and submit your "Accept" recommendation.

Reviewer #1: All comments have been addressed

Reviewer #3: (No Response)

 2. Is the manuscript technically sound, and do the data support the conclusions?

The manuscript must describe a technically sound piece of scientific research with data that supports the conclusions. Experiments must have been conducted rigorously, with appropriate controls, replication, and sample sizes. The conclusions must be drawn appropriately based on the data presented.

Reviewer #1: Partly

Reviewer #3: (No Response)

 3. Has the statistical analysis been performed appropriately and rigorously?

Reviewer #1: I Don't Know

Reviewer #3: (No Response)

 4. Have the authors made all data underlying the findings in their manuscript fully available?

The [PLOS Data policy](http://track.editorialmanager.com/CL0/http:%2F%2Fwww.plosone.org%2Fstatic%2Fpolicies.action%23sharing/1/010f019cf50ac5ee-f1ce14aa-09ce-466f-9071-9edd36d4236e-000000/uF2dxM4gN4Z_PK5oK7FG7aznl6w7v0RwUvdIHf1fng4=254) requires authors to make all data underlying the findings described in their manuscript fully available without restriction, with rare exception (please refer to the Data Availability Statement in the manuscript PDF file). The data should be provided as part of the manuscript or its supporting information, or deposited to a public repository. For example, in addition to summary statistics, the data points behind means, medians and variance measures should be available. If there are restrictions on publicly sharing data—e.g. participant privacy or use of data from a third party—those must be specified.

Reviewer #1: Yes

Reviewer #3: (No Response)

 5. Is the manuscript presented in an intelligible fashion and written in standard English?

PLOS ONE does not copyedit accepted manuscripts, so the language in submitted articles must be clear, correct, and unambiguous. Any typographical or grammatical errors should be corrected at revision, so please note any specific errors here.

Reviewer #1: Yes

Reviewer #3: Yes

 6. Review Comments to the Author

Please use the space provided to explain your answers to the questions above. You may also include additional comments for the author, including concerns about dual publication, research ethics, or publication ethics. (Please upload your review as an attachment if it exceeds 20,000 characters)

Reviewer #1: Thanks for making essetial edits to the article "Enhancing the long-term abstinence rate of smoking through telemedicine: A multicenter, retrospective cohort study in Japan"

Reviewer #3: The authors have not yet corrected the symbols in the tables. There is a difference between ≥ and >; for example, ≥ 700 includes 700, whereas > 700 does not.

Tables to be corrected:
Table 1: ≥700 and ≥75
Table 3: ≥ 45, ≥ 25. 4 days and above in a week, ≥ 700, ≥ 75
Table 5: ≥ 45, ≥ 25, 4 days and above in a week, ≥ 75

It is recommended that the authors have the tables/manuscript proofread by another person.

 7. PLOS authors have the option to publish the peer review history of their article ([what does this mean?](https://track.editorialmanager.com/CL0/https:%2F%2Fjournals.plos.org%2Fplosone%2Fs%2Feditorial-and-peer-review-process%23loc-peer-review-history/1/010f019cf50ac5ee-f1ce14aa-09ce-466f-9071-9edd36d4236e-000000/BGZmF3ow15Yz4Q3MCC-nrPBrk275KyB3YXDFlKHkDAE=254)). If published, this will include your full peer review and any attached files.

If you choose “no”, your identity will remain anonymous but your review may still be made public.

**Do you want your identity to be public for this peer review?** For information about this choice, including consent withdrawal, please see our [Privacy Policy](https://track.editorialmanager.com/CL0/https:%2F%2Fwww.plos.org%2Fprivacy-policy/1/010f019cf50ac5ee-f1ce14aa-09ce-466f-9071-9edd36d4236e-000000/LpyNv__VavA1T6x0w4_ne2w_LpiSlLMhMrL7Xt0ojHw=254).

Reviewer #1: **Yes:** Arun Prasad

Reviewer #3: No

→Table 1, 3, 5 were corrected properly in response to reviewers’ comments.

Sincerely,

Kazuki Takakura, M.D., Ph. D.

UnMed Clinic Motomachi

3-116 Motomachi, Naka-ku, Yokohama, Kanagawa 231-0861, Japan

E-mail: ktakakura@jikei.ac.jp
