# Supplementary material for: Enhancing the long-term abstinence rate of smoking through telemedicine: A multicenter, retrospective cohort study in Japan
Source: PLoS One. 2026 Jul 22;21(7):e0331514. doi: 10.1371/journal.pone.0331514 (PMC13390940; doi:10.1371/journal.pone.0331514)
Supplement: Attachment — Submitted filename: Response to Reviewers.(4) docx.docx [file pone.0331514.s005.docx]

**Response to Reviewers**

May 1, 2026

Dear Editor,

Thank you for giving me the opportunity to submit a revised draft of my manuscript titled **“Enhancing the long-term abstinence rate of smoking through telemedicine: A multicenter, retrospective cohort study in Japan”** to *PLOS ONE*. We appreciate the time and effort that you and the reviewers have dedicated to providing your feedback on my manuscript.

Journal Requirements:

1. If the reviewer comments include a recommendation to cite specific previously published works, please review and evaluate these publications to determine whether they are relevant and should be cited. There is no requirement to cite these works unless the editor has indicated otherwise.

→OK
2. Please review your reference list to ensure that it is complete and correct. If you have cited papers that have been retracted, please include the rationale for doing so in the manuscript text, or remove these references and replace them with relevant current references. Any changes to the reference list should be mentioned in the rebuttal letter that accompanies your revised manuscript. If you need to cite a retracted article, indicate the article’s retracted status in the References list and also include a citation and full reference for the retraction notice.

→OK

Additional Editor Comments :
Dear Author,
Please address the reviewer(s) comments.
→OK

Reviewers' comments:
Reviewer's Responses to Questions

**Comments to the Author**

1. If the authors have adequately addressed your comments raised in a previous round of review and you feel that this manuscript is now acceptable for publication, you may indicate that here to bypass the “Comments to the Author” section, enter your conflict of interest statement in the “Confidential to Editor” section, and submit your "Accept" recommendation.

Reviewer #4: All comments have been addressed

2. Is the manuscript technically sound, and do the data support the conclusions?

The manuscript must describe a technically sound piece of scientific research with data that supports the conclusions. Experiments must have been conducted rigorously, with appropriate controls, replication, and sample sizes. The conclusions must be drawn appropriately based on the data presented.

Reviewer #4: Yes

3. Has the statistical analysis been performed appropriately and rigorously?

Reviewer #4: Yes

4. Have the authors made all data underlying the findings in their manuscript fully available?

The [PLOS Data policy](http://track.editorialmanager.com/CL0/http:%2F%2Fwww.plosone.org%2Fstatic%2Fpolicies.action%23sharing/1/010f019da8706c46-0fc16b61-71bb-4a33-b362-eef88c3f033d-000000/01SVYR9tcNbthjBibTmeDkFPBSn0LUVZmcmo3driewg=258) requires authors to make all data underlying the findings described in their manuscript fully available without restriction, with rare exception (please refer to the Data Availability Statement in the manuscript PDF file). The data should be provided as part of the manuscript or its supporting information, or deposited to a public repository. For example, in addition to summary statistics, the data points behind means, medians and variance measures should be available. If there are restrictions on publicly sharing data—e.g. participant privacy or use of data from a third party—those must be specified.

Reviewer #4: Yes

5. Is the manuscript presented in an intelligible fashion and written in standard English?

PLOS ONE does not copyedit accepted manuscripts, so the language in submitted articles must be clear, correct, and unambiguous. Any typographical or grammatical errors should be corrected at revision, so please note any specific errors here.

Reviewer #4: Yes

6. Review Comments to the Author

Please use the space provided to explain your answers to the questions above. You may also include additional comments for the author, including concerns about dual publication, research ethics, or publication ethics. (Please upload your review as an attachment if it exceeds 20,000 characters)

Reviewer #4: Line 173: A total of 1514 + 1202 is more than the total of 3708 patients who participated in the smoking cessation program.
Lines from 181 to 184 are repeated.

→Thank you for the comments. As you pointed out, the numbers in the text were incorrect and, lines from 181 to 184 were repeated. Therefore, we have made the necessary corrections to the points you mentioned.

7. PLOS authors have the option to publish the peer review history of their article. If published, this will include your full peer review and any attached files.

If you choose “no”, your identity will remain anonymous but your review may still be made public.

**Do you want your identity to be public for this peer review?** For information about this choice, including consent withdrawal, please see our [Privacy Policy](https://track.editorialmanager.com/CL0/https:%2F%2Fwww.plos.org%2Fprivacy-policy/1/010f019da8706c46-0fc16b61-71bb-4a33-b362-eef88c3f033d-000000/kEWJ204TcP2sdUySyAUPmycJWB77lEWDEgXove6knHA=258).

Reviewer #4: **Yes:** ABDULRAZZAQ YAHYA AHMED AL-KHAZZAN

Sincerely,

Kazuki Takakura, M.D., Ph. D.

UnMed Clinic Motomachi

3-116 Motomachi, Naka-ku, Yokohama, Kanagawa 231-0861, Japan

E-mail: ktakakura@jikei.ac.jp
