# Supplementary material for: Enhancing the long-term abstinence rate of smoking through telemedicine: A multicenter, retrospective cohort study in Japan
Source: PLoS One. 2026 Jul 22;21(7):e0331514. doi: 10.1371/journal.pone.0331514 (PMC13390940; doi:10.1371/journal.pone.0331514)
Supplement: Peer review history

# Peer review: Decision Letter 0

**DOI:** 10.1371/journal.pone.0331514.r001

**Authors:** Saeed Ahmed

24 Sep 2025

PONE-D-25-20021

Enhancing the long-term abstinence rate of smoking through telemedicine: A multicenter, retrospective cohort study in Japan

PLOS ONE

Dear Dr. Takakura,

Thank you for submitting your manuscript to PLOS ONE. After careful consideration, we feel that it has merit but does not fully meet PLOS ONE’s publication criteria as it currently stands. Therefore, we invite you to submit a revised version of the manuscript that addresses the points raised during the review process.

Please submit your revised manuscript by Nov 08 2025 11:59PM. If you will need more time than this to complete your revisions, please reply to this message or contact the journal office at plosone@plos.org. When you're ready to submit your revision, log on to https://www.editorialmanager.com/pone/ and select the 'Submissions Needing Revision' folder to locate your manuscript file.

Please include the following items when submitting your revised manuscript:

- A rebuttal letter that responds to each point raised by the academic editor and reviewer(s). You should upload this letter as a separate file labeled 'Response to Reviewers'.
- A marked-up copy of your manuscript that highlights changes made to the original version. You should upload this as a separate file labeled 'Revised Manuscript with Track Changes'.
- An unmarked version of your revised paper without tracked changes. You should upload this as a separate file labeled 'Manuscript'.

If you would like to make changes to your financial disclosure, please include your updated statement in your cover letter. Guidelines for resubmitting your figure files are available below the reviewer comments at the end of this letter.

If applicable, we recommend that you deposit your laboratory protocols in protocols.io to enhance the reproducibility of your results. Protocols.io assigns your protocol its own identifier (DOI) so that it can be cited independently in the future. For instructions see: https://journals.plos.org/plosone/s/submission-guidelines#loc-laboratory-protocols. Additionally, PLOS ONE offers an option for publishing peer-reviewed Lab Protocol articles, which describe protocols hosted on protocols.io. Read more information on sharing protocols at https://plos.org/protocols?utm_medium=editorial-email&utm_source=authorletters&utm_campaign=protocols.

We look forward to receiving your revised manuscript.

Kind regards,

Saeed Ahmed, MD, FAPA, FASAM

Academic Editor

PLOS ONE

Journal Requirements:

When submitting your revision, we need you to address these additional requirements.

1. Please ensure that your manuscript meets PLOS ONE's style requirements, including those for file naming. The PLOS ONE style templates can be found at

https://journals.plos.org/plosone/s/file?id=wjVg/PLOSOne_formatting_sample_main_body.pdf and

https://journals.plos.org/plosone/s/file?id=ba62/PLOSOne_formatting_sample_title_authors_affiliations.pdf

2. Your ethics statement should only appear in the Methods section of your manuscript. If your ethics statement is written in any section besides the Methods, please delete it from any other section.

3. Please provide a complete Data Availability Statement in the submission form, ensuring you include all necessary access information or a reason for why you are unable to make your data freely accessible. If your research concerns only data provided within your submission, please write "All data are in the manuscript and/or supporting information files" as your Data Availability Statement.

4. If the reviewer comments include a recommendation to cite specific previously published works, please review and evaluate these publications to determine whether they are relevant and should be cited. There is no requirement to cite these works unless the editor has indicated otherwise.

Additional Editor Comments:

Your manuscript has been reviewed by our referees, who have provided valuable comments and suggestions. Please see the detailed feedback below to help you revise and improve your paper.

Reviewer #1: Attached is the PDF with suggested edits.

A few other limitations ti consider-

- Participants were self-selected individuals who applied for a telemedicine smoking cessation program via their health insurance association. This may lead to a population with higher motivation or resources for quitting smoking, which limits generalizability to all smokers.

- Data were collected from 20 medical institutions with widely varying sample sizes.

Variability in patient volume and potentially in clinical practices between sites could introduce site-level confounding or bias.

- Data such as smoking history, self-efficacy, and depressive symptoms were based on self-reported questionnaires.

This could lead to recall bias and misclassification.

-1,297 patients were excluded due to censoring (unclear follow-up status, lost to follow-up)

This could result in attrition bias.

-The cohort consisted of Japanese workers participating in a health-insurance-linked program, with most joining via their business sites. Findings may not generalize to unemployed individuals, non-Japanese populations, or those without access to similar healthcare systems or programs.

Reviewer #2: Thank you for the opportunity to review the manuscript which addresses a clinically important topic and contributes valuable insights into the comparative effectiveness of telemedicine vs face to face intervention in smoking cessation. Smoking is a major public health problem worldwide and addressing this issues and the investigation into telemedicine-based smoking cessation aligns with global shift in healthcare delivery, specially post COVID. The inclusion of over 3700 participants across multiple sites enhances the generalizability of findings with in the Japanese healthcare context. Clear primary and secondary outcomes, comparison with data from japan's MHLW adds contextual strengths and relevance. Authors have acknowledged potential selection bias, please clarify how patients were assigned face to face vs telemedicine on the basis of their preference, clinic assignment or another factor?. The manuscript contains frequent grammatical errors, would recommend proof reading. In intervention, consider including more details about the content and format of the 4 telemedicine sessions. Were there any specific modality of therapy, CBT, MI was utilized

Reviewer #3: The manuscript requires improvement.

The abstract needs to state how many participants for face-to-face. The results should highlight both telemedicine and face-to-face.

Line 46: Technically, the p-value cannot be zero (to use the symbol p < )

Table 1: 40 > to be revised to >40. Likewise for others 700 > and 75 >4 (to use ≤ or ≥ where applicable)

Table 1: Face-to-face data is to be included for comparison.

Table 3: 45< , > 25, 700 >, 75 > symbol used incorrectly (to use ≤ or ≥ where applicable). Figure 1: The figure requires revision to show the face-to-face arm.

Line 161-174: The section is to be divided according to telemedicine and face-to-face.

Table 2: Face-to-face data to be presented along with telemedicine data.

Line 184: To indicate the p-values for the comparison between completed vs not completed and abstinence vs non-abstinence.

Table 3: Separate analyses to be provided for telemedicine and face-to-face. Likewise, for Table 5.

Line 202: (P 0.000)?

Table 5: 45 <, > 25, over 4 days a week, 75 >, incorrect symbol was used. To use ≤ or ≥ where applicable. P-value is to be presented.

Please remove the underline for the confidence interval value, since the p-value is sufficient to indicate statistical significance. Similarly, the odds ratios (OR) should not be in bold.

For the adjustment analyses, the words adjusted and aOR are to be used where appropriate, including tables.

The fulfilment of the statistical tests' assumptions is to be stated e.g multicollinearity etc.

Model fit etc for logistic regression is to be presented.

[Note: HTML markup is below. Please do not edit.]

Reviewers' comments:

Reviewer's Responses to Questions

**Comments to the Author**

1. Is the manuscript technically sound, and do the data support the conclusions?

The manuscript must describe a technically sound piece of scientific research with data that supports the conclusions. Experiments must have been conducted rigorously, with appropriate controls, replication, and sample sizes. The conclusions must be drawn appropriately based on the data presented.

Reviewer #1: Partly

Reviewer #2: Yes

Reviewer #3: Partly

**********

2. Has the statistical analysis been performed appropriately and rigorously?

Reviewer #1: I Don't Know

Reviewer #2: Yes

Reviewer #3: No

**********

3. Have the authors made all data underlying the findings in their manuscript fully available?

The PLOS Data policy requires authors to make all data underlying the findings described in their manuscript fully available without restriction, with rare exception (please refer to the Data Availability Statement in the manuscript PDF file). The data should be provided as part of the manuscript or its supporting information, or deposited to a public repository. For example, in addition to summary statistics, the data points behind means, medians and variance measures should be available. If there are restrictions on publicly sharing data—e.g. participant privacy or use of data from a third party—those must be specified.

Reviewer #1: Yes

Reviewer #2: Yes

Reviewer #3: Yes

**********

4. Is the manuscript presented in an intelligible fashion and written in standard English?

PLOS ONE does not copyedit accepted manuscripts, so the language in submitted articles must be clear, correct, and unambiguous. Any typographical or grammatical errors should be corrected at revision, so please note any specific errors here.

Reviewer #1: Yes

Reviewer #2: Yes

Reviewer #3: Yes

**********

5. Review Comments to the Author

Please use the space provided to explain your answers to the questions above. You may also include additional comments for the author, including concerns about dual publication, research ethics, or publication ethics. (Please upload your review as an attachment if it exceeds 20,000 characters)

Reviewer #1: Attached is the PDF with suggested edits.

A few other limitations ti consider-

- Participants were self-selected individuals who applied for a telemedicine smoking cessation program via their health insurance association. This may lead to a population with higher motivation or resources for quitting smoking, which limits generalizability to all smokers.

- Data were collected from 20 medical institutions with widely varying sample sizes.

Variability in patient volume and potentially in clinical practices between sites could introduce site-level confounding or bias.

- Data such as smoking history, self-efficacy, and depressive symptoms were based on self-reported questionnaires.

This could lead to recall bias and misclassification.

-1,297 patients were excluded due to censoring (unclear follow-up status, lost to follow-up)

This could result in attrition bias.

-The cohort consisted of Japanese workers participating in a health-insurance-linked program, with most joining via their business sites. Findings may not generalize to unemployed individuals, non-Japanese populations, or those without access to similar healthcare systems or programs.

Reviewer #2: Thank you for the opportunity to review the manuscript which addresses a clinically important topic and contributes valuable insights into the comparative effectiveness of telemedicine vs face to face intervention in smoking cessation. Smoking is a major public health problem worldwide and addressing this issues and the investigation into telemedicine-based smoking cessation aligns with global shift in healthcare delivery, specially post COVID. The inclusion of over 3700 participants across multiple sites enhances the generalizability of findings with in the Japanese healthcare context. Clear primary and secondary outcomes, comparison with data from japan's MHLW adds contextual strengths and relevance. Authors have acknowledged potential selection bias, please clarify how patients were assigned face to face vs telemedicine on the basis of their preference, clinic assignment or another factor?. The manuscript contains frequent grammatical errors, would recommend proof reading. In intervention, consider including more details about the content and format of the 4 telemedicine sessions. Were there any specific modality of therapy, CBT, MI was utilized. Overall, this is a well-conducted large scale multi-center study that offers strong evidence supporting the use of telemedicine for smoking cessation.

Reviewer #3: The manuscript requires improvement.

The abstract needs to state how many participants for face-to-face. The results should highlight both telemedicine and face-to-face.

Line 46: Technically, the p-value cannot be zero (to use the symbol p < )

Table 1: 40 > to be revised to >40. Likewise for others 700 > and 75 >4 (to use ≤ or ≥ where applicable)

Table 1: Face-to-face data is to be included for comparison.

Table 3: 45< , > 25, 700 >, 75 > symbol used incorrectly (to use ≤ or ≥ where applicable). Figure 1: The figure requires revision to show the face-to-face arm.

Line 161-174: The section is to be divided according to telemedicine and face-to-face.

Table 2: Face-to-face data to be presented along with telemedicine data.

Line 184: To indicate the p-values for the comparison between completed vs not completed and abstinence vs non-abstinence.

Table 3: Separate analyses to be provided for telemedicine and face-to-face. Likewise, for Table 5.

Line 202: (P 0.000)?

Table 5: 45 <, > 25, over 4 days a week, 75 >, incorrect symbol was used. To use ≤ or ≥ where applicable. P-value is to be presented.

Please remove the underline for the confidence interval value, since the p-value is sufficient to indicate statistical significance. Similarly, the odds ratios (OR) should not be in bold.

For the adjustment analyses, the words adjusted and aOR are to be used where appropriate, including tables.

The fulfilment of the statistical tests' assumptions is to be stated e.g multicollinearity etc.

Model fit etc for logistic regression is to be presented.

**********

6. PLOS authors have the option to publish the peer review history of their article (what does this mean?). If published, this will include your full peer review and any attached files.

If you choose “no”, your identity will remain anonymous but your review may still be made public.

**Do you want your identity to be public for this peer review?**  For information about this choice, including consent withdrawal, please see our Privacy Policy.

Reviewer #1: **Yes:** Arun Prasad

Reviewer #2: **Yes:** Rajesh Mehta

Reviewer #3: No

**********

[NOTE: If reviewer comments were submitted as an attachment file, they will be attached to this email and accessible via the submission site. Please log into your account, locate the manuscript record, and check for the action link "View Attachments". If this link does not appear, there are no attachment files.]

While revising your submission, please upload your figure files to the Preflight Analysis and Conversion Engine (PACE) digital diagnostic tool, https://pacev2.apexcovantage.com/. PACE helps ensure that figures meet PLOS requirements. To use PACE, you must first register as a user. Registration is free. Then, login and navigate to the UPLOAD tab, where you will find detailed instructions on how to use the tool. If you encounter any issues or have any questions when using PACE, please email PLOS at figures@plos.org. Please note that Supporting Information files do not need this step.

Attachment Submitted filename: Completed - PONE-D-25-20021_reviewer.pdf

# Author comment: Author response to Decision Letter 1

**DOI:** 10.1371/journal.pone.0331514.r002

27 Oct 2025

Response to Reviewers

October 26, 2025

Dear Editor,

Thank you for giving me the opportunity to submit a revised draft of my manuscript titled “Enhancing the long-term abstinence rate of smoking through telemedicine: A multicenter, retrospective cohort study in Japan” to PLOS ONE. We appreciate the time and effort that you and the reviewers have dedicated to providing your valuable feedback on my manuscript. We are grateful to the reviewers for their insightful comments on my paper. We have been able to incorporate changes to reflect most of the suggestions provided by the reviewers. Here is a point-by-point response to the reviewers’ comments and concerns.

Comments from Journal

When submitting your revision, we need you to address these additional requirements.

1. Please ensure that your manuscript meets PLOS ONE's style requirements, including those for file naming. The PLOS ONE style templates can be found at

https://journals.plos.org/plosone/s/file?id=wjVg/PLOSOne_formatting_sample_main_body.pdf and

https://journals.plos.org/plosone/s/file?id=ba62/PLOSOne_formatting_sample_title_authors_affiliations.pdf

→ We tried for our manuscript to meet your style requirements.

2. Your ethics statement should only appear in the Methods section of your manuscript. If your ethics statement is written in any section besides the Methods, please delete it from any other section.

→We deleted the statement from any other section.

3. Please provide a complete Data Availability Statement in the submission form, ensuring you include all necessary access information or a reason for why you are unable to make your data freely accessible. If your research concerns only data provided within your submission, please write "All data are in the manuscript and/or supporting information files" as your Data Availability Statement.

→ We provided a complete Data Availability Statement in the submission form.

4. If the reviewer comments include a recommendation to cite specific previously published works, please review and evaluate these publications to determine whether they are relevant and should be cited. There is no requirement to cite these works unless the editor has indicated otherwise.

→ We got it.

Comments from Reviewer 1

- Participants were self-selected individuals who applied for a telemedicine smoking cessation program via their health insurance association. This may lead to a population with higher motivation or resources for quitting smoking, which limits generalizability to all smokers.

-The cohort consisted of Japanese workers participating in a health-insurance-linked program, with most joining via their business sites. Findings may not generalize to unemployed individuals, non-Japanese populations, or those without access to similar healthcare systems or programs.

Response: Thank you for the comments.

We understood that the points raised in these two comments are largely similar. We agree with the comments. Therefore, we added the explanation as a limitation in Discussion section.

- Data were collected from 20 medical institutions with widely varying sample sizes.

Variability in patient volume and potentially in clinical practices between sites could introduce site-level confounding or bias.

Response: Thank you for pointing this out. However, the attributes of all of the participating medical institutes of this study are consistent as a clinic, not large hospital. It is not unusual that there are a lot of low-volume clinics, on the basis of a statistical result showing that average number of patients who want to quit smoking per facility per year is 12.8. In addition, a certain level of medical skill is guaranteed in the program, because all of the attending doctors basically completed the required online learning about smoking cessation.

- Data such as smoking history, self-efficacy, and depressive symptoms were based on self-reported questionnaires. This could lead to recall bias and misclassification.

-1,297 patients were excluded due to censoring (unclear follow-up status, lost to follow-up)

This could result in attrition bias.

Response: Thank you for the comments.

We agree with both of the comments. We have accordingly added the sentences as limitations in Discussion section.

Comments from Reviewer 2

Thank you for the opportunity to review the manuscript which addresses a clinically important topic and contributes valuable insights into the comparative effectiveness of telemedicine vs face to face intervention in smoking cessation. Smoking is a major public health problem worldwide and addressing this issues and the investigation into telemedicine-based smoking cessation aligns with global shift in healthcare delivery, specially post COVID. The inclusion of over 3700 participants across multiple sites enhances the generalizability of findings with in the Japanese healthcare context. Clear primary and secondary outcomes, comparison with data from japan's MHLW adds contextual strengths and relevance.

Response: Thank you for your positive feedback.

Authors have acknowledged potential selection bias, please clarify how patients were assigned face to face vs telemedicine on the basis of their preference, clinic assignment or another factor?

Response: Thank you for pointing this out. However, as mentioned in Materials and methods section, we just provided the online program only. And a comparison target of face-to-face was prepared from the database of the Ministry of Health, Labor and Welfare (MHLW) in Japan. Therefore, all of the included patient made decisions based on their own will.

The manuscript contains frequent grammatical errors, would recommend proof reading.

Response: Thank you for the comments.

Actually, we have used an editing service by a native English speaker for this manuscript. I attached the certificate.

In intervention, consider including more details about the content and format of the 4 telemedicine sessions. Were there any specific modality of therapy, CBT, MI was utilized

Response: Thank you for the comments.

Figure 2 shows in detail about the online program with 4 telemedicine sessions. All of the attending doctors basically completed the required online learning about smoking cessation counseling and approach the smoking cessation program with understanding the cognitive behavioral therapy. Therefore, all of the patients were treated similarly without any specific therapeutic modality in the program.

Comments from Reviewer 3

The abstract needs to state how many participants for face-to-face. The results should highlight both telemedicine and face-to-face.

Response: Thank you for pointing this out.

We agree with this comment. We have accordingly modified the sentences in Abstract.

Line 46: Technically, the p-value cannot be zero (to use the symbol p < )

Response: Thank you for the comments.

We modified the P value to <0.005.

Table 1: 40 > to be revised to >40. Likewise for others 700 > and 75 >4 (to use ≤ or ≥ where applicable)

Response: Thank you for the comments.

We corrected the point in Table 1.

Table 1: Face-to-face data is to be included for comparison.

Response: Thank you for the comments.

However, it is difficult to include face-to-face data in Table 1. Because these targeted patients are different with online and face-to-face. Instead, we gave a supplementary explanation about that in Results section.

Table 3: 45< , > 25, 700 >, 75 > symbol used incorrectly (to use ≤ or ≥ where applicable). Response: Thank you for the comments.

We corrected the point in Table 3.

Figure 1: The figure requires revision to show the face-to-face arm.

Response: Thank you for the comments.

However, Figure 1 just showed the diagram of the patients treated from us through online. Then, we didn’t treat for the patients of the face-to-face group. Therefore, we modified the Figure legends appropriately.

Line 161-174: The section is to be divided according to telemedicine and face-to-face.

Response: Thank you for pointing this out.

We agree with this comment. We have accordingly added explanation for face-to-face group in the section.

Table 2: Face-to-face data to be presented along with telemedicine data.

Response: Thank you for the comments.

However, it is too complicated if face-to-face data is also demonstrated in Table 2. Because the treatment duration with varenicline is 12 weeks in face-to-face examination (4 weeks longer than online examination), even though the treatment duration with nicotine patch is same.

Line 184: To indicate the p-values for the comparison between completed vs not completed and abstinence vs non-abstinence.

Response: Thank you for the comments.

Unfortunately, there is no data available for the p-values for the comparison from the Ministry of Health, Labor and Welfare in Japan.

Table 3: Separate analyses to be provided for telemedicine and face-to-face. Likewise, for Table 5.

Response: Thank you for the comments.

Unfortunately, there is no data available for the specific related factors of face-to-face examinations from the Ministry of Health, Labor and Welfare in Japan.

Line 202: (P 0.000)?

Response: Thank you for the comments.

We modified the P value to <0.001.

Table 5: 45 <, > 25, over 4 days a week, 75 >, incorrect symbol was used. To use ≤ or ≥ where applicable. P-value is to be presented.

Response: Thank you for the comments.

We corrected the point in Table 5 added the p-value.

Please remove the underline for the confidence interval value, since the p-value is sufficient to indicate statistical significance. Similarly, the odds ratios (OR) should not be in bold.

For the adjustment analyses, the words adjusted and aOR are to be used where appropriate, including tables.

The fulfilment of the statistical tests' assumptions is to be stated e.g multicollinearity etc.

Model fit etc for logistic regression is to be presented.

Response: Thank you for the comments.

We corrected the points in all of the Tables.

In addition, the fulfilment of the statistical test’s assumptions is stated in “Statistical analysis” section.

Sincerely,

Kazuki Takakura, M.D., Ph. D.

UnMed Clinic Motomachi

3-116 Motomachi, Naka-ku, Yokohama, Kanagawa 231-0861, Japan

E-mail: ktakakura@jikei.ac.jp

Attachment Submitted filename: Response to Reviewers.docx

# Peer review: Decision Letter 1

**DOI:** 10.1371/journal.pone.0331514.r003

**Authors:** Saeed Ahmed

7 Jan 2026

PONE-D-25-20021R1

Enhancing the long-term abstinence rate of smoking through telemedicine: A multicenter, retrospective cohort study in Japan

PLOS One

Dear Dr. Takakura,

Thank you for submitting your manuscript to PLOS ONE. After careful consideration, we feel that it has merit but does not fully meet PLOS ONE’s publication criteria as it currently stands. Therefore, we invite you to submit a revised version of the manuscript that addresses the points raised during the review process.

Please submit your revised manuscript by Feb 21 2026 11:59PM. If you will need more time than this to complete your revisions, please reply to this message or contact the journal office at plosone@plos.org. When you're ready to submit your revision, log on to https://www.editorialmanager.com/pone/ and select the 'Submissions Needing Revision' folder to locate your manuscript file.

Please include the following items when submitting your revised manuscript:

- A letter that responds to each point raised by the academic editor and reviewer(s). You should upload this letter as a separate file labeled 'Response to Reviewers'.
- A marked-up copy of your manuscript that highlights changes made to the original version. You should upload this as a separate file labeled 'Revised Manuscript with Track Changes'.
- An unmarked version of your revised paper without tracked changes. You should upload this as a separate file labeled 'Manuscript'.

-->If you would like to make changes to your financial disclosure, please include your updated statement in your cover letter. Guidelines for resubmitting your figure files are available below the reviewer comments at the end of this letter.

If applicable, we recommend that you deposit your laboratory protocols in protocols.io to enhance the reproducibility of your results. Protocols.io assigns your protocol its own identifier (DOI) so that it can be cited independently in the future. For instructions see: https://journals.plos.org/plosone/s/submission-guidelines#loc-laboratory-protocols. Additionally, PLOS ONE offers an option for publishing peer-reviewed Lab Protocol articles, which describe protocols hosted on protocols.io. Read more information on sharing protocols at https://plos.org/protocols?utm_medium=editorial-email&utm_source=authorletters&utm_campaign=protocols.

We look forward to receiving your revised manuscript.

Kind regards,

Saeed Ahmed, MD

Academic Editor

PLOS One

Journal Requirements:

If the reviewer comments include a recommendation to cite specific previously published works, please review and evaluate these publications to determine whether they are relevant and should be cited. There is no requirement to cite these works unless the editor has indicated otherwise.

Please review your reference list to ensure that it is complete and correct. If you have cited papers that have been retracted, please include the rationale for doing so in the manuscript text, or remove these references and replace them with relevant current references. Any changes to the reference list should be mentioned in the rebuttal letter that accompanies your revised manuscript. If you need to cite a retracted article, indicate the article’s retracted status in the References list and also include a citation and full reference for the retraction notice.

[Note: HTML markup is below. Please do not edit.]

Reviewers' comments:

Reviewer's Responses to Questions

**Comments to the Author**

1. If the authors have adequately addressed your comments raised in a previous round of review and you feel that this manuscript is now acceptable for publication, you may indicate that here to bypass the “Comments to the Author” section, enter your conflict of interest statement in the “Confidential to Editor” section, and submit your "Accept" recommendation.

Reviewer #1: All comments have been addressed

Reviewer #3: (No Response)

**********

2. Is the manuscript technically sound, and do the data support the conclusions?

The manuscript must describe a technically sound piece of scientific research with data that supports the conclusions. Experiments must have been conducted rigorously, with appropriate controls, replication, and sample sizes. The conclusions must be drawn appropriately based on the data presented.

Reviewer #1: Yes

Reviewer #3: Partly

**********

3. Has the statistical analysis been performed appropriately and rigorously?

Reviewer #1: I Don't Know

Reviewer #3: No

**********

4. Have the authors made all data underlying the findings in their manuscript fully available?

The PLOS Data policy requires authors to make all data underlying the findings described in their manuscript fully available without restriction, with rare exception (please refer to the Data Availability Statement in the manuscript PDF file). The data should be provided as part of the manuscript or its supporting information, or deposited to a public repository. For example, in addition to summary statistics, the data points behind means, medians and variance measures should be available. If there are restrictions on publicly sharing data—e.g. participant privacy or use of data from a third party—those must be specified.

Reviewer #1: Yes

Reviewer #3: (No Response)

**********

5. Is the manuscript presented in an intelligible fashion and written in standard English?

PLOS ONE does not copyedit accepted manuscripts, so the language in submitted articles must be clear, correct, and unambiguous. Any typographical or grammatical errors should be corrected at revision, so please note any specific errors here.

Reviewer #1: Yes

Reviewer #3: Yes

**********

6. Review Comments to the Author

Please use the space provided to explain your answers to the questions above. You may also include additional comments for the author, including concerns about dual publication, research ethics, or publication ethics. (Please upload your review as an attachment if it exceeds 20,000 characters)

Reviewer #1: Thank you for making the edits and responding to the queries on "Enhancing the long-term abstinence rate of smoking through telemedicine: A multicenter, retrospective cohort study in Japan". Best of luck on publication!

Reviewer #3: Please check the categories and symbols in the tables thoroughly.

I was unable to locate some of the changes in the manuscript that the authors indicated.

**********

7. PLOS authors have the option to publish the peer review history of their article (what does this mean?). If published, this will include your full peer review and any attached files.

If you choose “no”, your identity will remain anonymous but your review may still be made public.

**Do you want your identity to be public for this peer review?**  For information about this choice, including consent withdrawal, please see our Privacy Policy.

Reviewer #1: **Yes:** Arun Prasad

Reviewer #3: No

**********

[NOTE: If reviewer comments were submitted as an attachment file, they will be attached to this email and accessible via the submission site. Please log into your account, locate the manuscript record, and check for the action link "View Attachments". If this link does not appear, there are no attachment files.]

To ensure your figures meet our technical requirements, please review our figure guidelines: https://journals.plos.org/plosone/s/figures

You may also use PLOS’s free figure tool, NAAS, to help you prepare publication quality figures: https://journals.plos.org/plosone/s/figures#loc-tools-for-figure-preparation.

NAAS will assess whether your figures meet our technical requirements by comparing each figure against our figure specifications.

# Author comment: Author response to Decision Letter 2

**DOI:** 10.1371/journal.pone.0331514.r004

15 Jan 2026

Because we found that No.25 is not suited as a cited reference in our manuscript, we replaced with another article. In addition, all of the Tables in the previous revised manuscript was not corrected properly in response to reviewers’ comments. Therefore, we modified completely all of the Tables in this round.

Attachment Submitted filename: Response to Reviewers (2).docx

# Peer review: Decision Letter 2

**DOI:** 10.1371/journal.pone.0331514.r005

**Authors:** Ali Blebil

16 Mar 2026

PONE-D-25-20021R2

Enhancing the long-term abstinence rate of smoking through telemedicine: A multicenter, retrospective cohort study in Japan

PLOS One

Dear Dr. Takakura,

Thank you for submitting your manuscript to PLOS ONE. After careful consideration, we feel that it has merit but does not fully meet PLOS ONE’s publication criteria as it currently stands. Therefore, we invite you to submit a revised version of the manuscript that addresses the points raised during the review process.

Please submit your revised manuscript by Apr 27 2026 11:59PM. If you will need more time than this to complete your revisions, please reply to this message or contact the journal office at plosone@plos.org. When you're ready to submit your revision, log on to https://www.editorialmanager.com/pone/ and select the 'Submissions Needing Revision' folder to locate your manuscript file.

Please include the following items when submitting your revised manuscript:

- A letter that responds to each point raised by the academic editor and reviewer(s). You should upload this letter as a separate file labeled 'Response to Reviewers'.
- A marked-up copy of your manuscript that highlights changes made to the original version. You should upload this as a separate file labeled 'Revised Manuscript with Track Changes'.
- An unmarked version of your revised paper without tracked changes. You should upload this as a separate file labeled 'Manuscript'.

-->If you would like to make changes to your financial disclosure, please include your updated statement in your cover letter. Guidelines for resubmitting your figure files are available below the reviewer comments at the end of this letter.

If applicable, we recommend that you deposit your laboratory protocols in protocols.io to enhance the reproducibility of your results. Protocols.io assigns your protocol its own identifier (DOI) so that it can be cited independently in the future. For instructions see: https://journals.plos.org/plosone/s/submission-guidelines#loc-laboratory-protocols. Additionally, PLOS ONE offers an option for publishing peer-reviewed Lab Protocol articles, which describe protocols hosted on protocols.io. Read more information on sharing protocols at https://plos.org/protocols?utm_medium=editorial-email&utm_source=authorletters&utm_campaign=protocols.

We look forward to receiving your revised manuscript.

Kind regards,

Ali Blebil, Ph.D.

Academic Editor

PLOS One

Journal Requirements:

1. If the reviewer comments include a recommendation to cite specific previously published works, please review and evaluate these publications to determine whether they are relevant and should be cited. There is no requirement to cite these works unless the editor has indicated otherwise.

2. Please review your reference list to ensure that it is complete and correct. If you have cited papers that have been retracted, please include the rationale for doing so in the manuscript text, or remove these references and replace them with relevant current references. Any changes to the reference list should be mentioned in the rebuttal letter that accompanies your revised manuscript. If you need to cite a retracted article, indicate the article’s retracted status in the References list and also include a citation and full reference for the retraction notice.

[Note: HTML markup is below. Please do not edit.]

Reviewer's Responses to Questions

**Comments to the Author**

1. If the authors have adequately addressed your comments raised in a previous round of review and you feel that this manuscript is now acceptable for publication, you may indicate that here to bypass the “Comments to the Author” section, enter your conflict of interest statement in the “Confidential to Editor” section, and submit your "Accept" recommendation.

Reviewer #1: All comments have been addressed

Reviewer #3: (No Response)

**********

2. Is the manuscript technically sound, and do the data support the conclusions?

The manuscript must describe a technically sound piece of scientific research with data that supports the conclusions. Experiments must have been conducted rigorously, with appropriate controls, replication, and sample sizes. The conclusions must be drawn appropriately based on the data presented.

Reviewer #1: Partly

Reviewer #3: (No Response)

**********

3. Has the statistical analysis been performed appropriately and rigorously?

Reviewer #1: I Don't Know

Reviewer #3: (No Response)

**********

4. Have the authors made all data underlying the findings in their manuscript fully available?

The PLOS Data policy requires authors to make all data underlying the findings described in their manuscript fully available without restriction, with rare exception (please refer to the Data Availability Statement in the manuscript PDF file). The data should be provided as part of the manuscript or its supporting information, or deposited to a public repository. For example, in addition to summary statistics, the data points behind means, medians and variance measures should be available. If there are restrictions on publicly sharing data—e.g. participant privacy or use of data from a third party—those must be specified.

Reviewer #1: Yes

Reviewer #3: (No Response)

**********

5. Is the manuscript presented in an intelligible fashion and written in standard English?

PLOS ONE does not copyedit accepted manuscripts, so the language in submitted articles must be clear, correct, and unambiguous. Any typographical or grammatical errors should be corrected at revision, so please note any specific errors here.

Reviewer #1: Yes

Reviewer #3: Yes

**********

6. Review Comments to the Author

Please use the space provided to explain your answers to the questions above. You may also include additional comments for the author, including concerns about dual publication, research ethics, or publication ethics. (Please upload your review as an attachment if it exceeds 20,000 characters)

Reviewer #1: Thanks for making essetial edits to the article "Enhancing the long-term abstinence rate of smoking through telemedicine: A multicenter, retrospective cohort study in Japan"

Reviewer #3: The authors have not yet corrected the symbols in the tables. There is a difference between ≥ and >; for example, ≥ 700 includes 700, whereas > 700 does not.

Tables to be corrected:

Table 1: ≥700 and ≥75

Table 3: ≥ 45, ≥ 25. 4 days and above in a week, ≥ 700, ≥ 75

Table 5: ≥ 45, ≥ 25, 4 days and above in a week, ≥ 75

It is recommended that the authors have the tables/manuscript proofread by another person.

**********

7. PLOS authors have the option to publish the peer review history of their article (what does this mean?). If published, this will include your full peer review and any attached files.

If you choose “no”, your identity will remain anonymous but your review may still be made public.

**Do you want your identity to be public for this peer review?**  For information about this choice, including consent withdrawal, please see our Privacy Policy.

Reviewer #1: **Yes:** Arun Prasad

Reviewer #3: No

**********

[NOTE: If reviewer comments were submitted as an attachment file, they will be attached to this email and accessible via the submission site. Please log into your account, locate the manuscript record, and check for the action link "View Attachments". If this link does not appear, there are no attachment files.]

To ensure your figures meet our technical requirements, please review our figure guidelines: https://journals.plos.org/plosone/s/figures

You may also use PLOS’s free figure tool, NAAS, to help you prepare publication quality figures: https://journals.plos.org/plosone/s/figures#loc-tools-for-figure-preparation.

NAAS will assess whether your figures meet our technical requirements by comparing each figure against our figure specifications.

# Author comment: Author response to Decision Letter 3

**DOI:** 10.1371/journal.pone.0331514.r006

18 Mar 2026

March 17, 2026

Dear Editor,

Thank you for giving me the opportunity to submit a revised draft of my manuscript titled “Enhancing the long-term abstinence rate of smoking through telemedicine: A multicenter, retrospective cohort study in Japan” to PLOS ONE. We appreciate the time and effort that you and the reviewers have dedicated to providing your feedback on my manuscript.

Reviewer's Responses to Questions

Comments to the Author

1. If the authors have adequately addressed your comments raised in a previous round of review and you feel that this manuscript is now acceptable for publication, you may indicate that here to bypass the “Comments to the Author” section, enter your conflict of interest statement in the “Confidential to Editor” section, and submit your "Accept" recommendation.

Reviewer #1: All comments have been addressed

Reviewer #3: (No Response)

________________________________________

2. Is the manuscript technically sound, and do the data support the conclusions?

The manuscript must describe a technically sound piece of scientific research with data that supports the conclusions. Experiments must have been conducted rigorously, with appropriate controls, replication, and sample sizes. The conclusions must be drawn appropriately based on the data presented.

Reviewer #1: Partly

Reviewer #3: (No Response)

________________________________________

3. Has the statistical analysis been performed appropriately and rigorously?

Reviewer #1: I Don't Know

Reviewer #3: (No Response)

________________________________________

4. Have the authors made all data underlying the findings in their manuscript fully available?

The PLOS Data policy requires authors to make all data underlying the findings described in their manuscript fully available without restriction, with rare exception (please refer to the Data Availability Statement in the manuscript PDF file). The data should be provided as part of the manuscript or its supporting information, or deposited to a public repository. For example, in addition to summary statistics, the data points behind means, medians and variance measures should be available. If there are restrictions on publicly sharing data—e.g. participant privacy or use of data from a third party—those must be specified.

Reviewer #1: Yes

Reviewer #3: (No Response)

________________________________________

5. Is the manuscript presented in an intelligible fashion and written in standard English?

PLOS ONE does not copyedit accepted manuscripts, so the language in submitted articles must be clear, correct, and unambiguous. Any typographical or grammatical errors should be corrected at revision, so please note any specific errors here.

Reviewer #1: Yes

Reviewer #3: Yes

________________________________________

6. Review Comments to the Author

Please use the space provided to explain your answers to the questions above. You may also include additional comments for the author, including concerns about dual publication, research ethics, or publication ethics. (Please upload your review as an attachment if it exceeds 20,000 characters)

Reviewer #1: Thanks for making essetial edits to the article "Enhancing the long-term abstinence rate of smoking through telemedicine: A multicenter, retrospective cohort study in Japan"

Reviewer #3: The authors have not yet corrected the symbols in the tables. There is a difference between ≥ and >; for example, ≥ 700 includes 700, whereas > 700 does not.

Tables to be corrected:

Table 1: ≥700 and ≥75

Table 3: ≥ 45, ≥ 25. 4 days and above in a week, ≥ 700, ≥ 75

Table 5: ≥ 45, ≥ 25, 4 days and above in a week, ≥ 75

It is recommended that the authors have the tables/manuscript proofread by another person.

________________________________________

7. PLOS authors have the option to publish the peer review history of their article (what does this mean?). If published, this will include your full peer review and any attached files.

If you choose “no”, your identity will remain anonymous but your review may still be made public.

Do you want your identity to be public for this peer review? For information about this choice, including consent withdrawal, please see our Privacy Policy.

Reviewer #1: Yes: Arun Prasad

Reviewer #3: No

→Table 1, 3, 5 were corrected properly in response to reviewers’ comments.

Sincerely,

Kazuki Takakura, M.D., Ph. D.

UnMed Clinic Motomachi

3-116 Motomachi, Naka-ku, Yokohama, Kanagawa 231-0861, Japan

E-mail: ktakakura@jikei.ac.jp

Attachment Submitted filename: Response to Reviewers.(3) docx.docx

# Peer review: Decision Letter 3

**DOI:** 10.1371/journal.pone.0331514.r007

**Authors:** Ali Blebil

19 Apr 2026

PONE-D-25-20021R3

Enhancing the long-term abstinence rate of smoking through telemedicine: A multicenter, retrospective cohort study in Japan

PLOS One

Dear Dr. Takakura,

Thank you for submitting your manuscript to PLOS ONE. After careful consideration, we feel that it has merit but does not fully meet PLOS ONE’s publication criteria as it currently stands. Therefore, we invite you to submit a revised version of the manuscript that addresses the points raised during the review process.

Please submit your revised manuscript by Jun 03 2026 11:59PM. If you will need more time than this to complete your revisions, please reply to this message or contact the journal office at plosone@plos.org. When you're ready to submit your revision, log on to https://www.editorialmanager.com/pone/ and select the 'Submissions Needing Revision' folder to locate your manuscript file.

Please include the following items when submitting your revised manuscript:

- A letter that responds to each point raised by the academic editor and reviewer(s). You should upload this letter as a separate file labeled 'Response to Reviewers'.
- A marked-up copy of your manuscript that highlights changes made to the original version. You should upload this as a separate file labeled 'Revised Manuscript with Track Changes'.
- An unmarked version of your revised paper without tracked changes. You should upload this as a separate file labeled 'Manuscript'.

If you would like to make changes to your financial disclosure, please include your updated statement in your cover letter. Guidelines for resubmitting your figure files are available below the reviewer comments at the end of this letter.

If applicable, we recommend that you deposit your laboratory protocols in protocols.io to enhance the reproducibility of your results. Protocols.io assigns your protocol its own identifier (DOI) so that it can be cited independently in the future. For instructions see: https://journals.plos.org/plosone/s/submission-guidelines#loc-laboratory-protocols. Additionally, PLOS ONE offers an option for publishing peer-reviewed Lab Protocol articles, which describe protocols hosted on protocols.io. Read more information on sharing protocols at https://plos.org/protocols?utm_medium=editorial-email&utm_source=authorletters&utm_campaign=protocols.

As the corresponding author, your ORCID iD is verified in the submission system and will appear in the published article. PLOS supports the use of ORCID, and we encourage all coauthors to register for an ORCID iD and use it as well. Please encourage your coauthors to verify their ORCID iD within the submission system before final acceptance, as unverified ORCID iDs will not appear in the published article. *Only*  the individual author can complete the verification step; PLOS staff *cannot*  verify ORCID iDs on behalf of authors.

We look forward to receiving your revised manuscript.

Kind regards,

Ali Blebil, Ph.D.

Academic Editor

PLOS One

Journal Requirements:

1. If the reviewer comments include a recommendation to cite specific previously published works, please review and evaluate these publications to determine whether they are relevant and should be cited. There is no requirement to cite these works unless the editor has indicated otherwise.

2. Please review your reference list to ensure that it is complete and correct. If you have cited papers that have been retracted, please include the rationale for doing so in the manuscript text, or remove these references and replace them with relevant current references. Any changes to the reference list should be mentioned in the rebuttal letter that accompanies your revised manuscript. If you need to cite a retracted article, indicate the article’s retracted status in the References list and also include a citation and full reference for the retraction notice.

Additional Editor Comments :

Dear Author,

Please address the reviewer(s) comments.

Reviewers' comments:

Reviewer's Responses to Questions

**Comments to the Author**

1. If the authors have adequately addressed your comments raised in a previous round of review and you feel that this manuscript is now acceptable for publication, you may indicate that here to bypass the “Comments to the Author” section, enter your conflict of interest statement in the “Confidential to Editor” section, and submit your "Accept" recommendation.

Reviewer #4: All comments have been addressed

**********

2. Is the manuscript technically sound, and do the data support the conclusions?

The manuscript must describe a technically sound piece of scientific research with data that supports the conclusions. Experiments must have been conducted rigorously, with appropriate controls, replication, and sample sizes. The conclusions must be drawn appropriately based on the data presented.

Reviewer #4: Yes

**********

3. Has the statistical analysis been performed appropriately and rigorously?

Reviewer #4: Yes

**********

4. Have the authors made all data underlying the findings in their manuscript fully available?

The PLOS Data policy requires authors to make all data underlying the findings described in their manuscript fully available without restriction, with rare exception (please refer to the Data Availability Statement in the manuscript PDF file). The data should be provided as part of the manuscript or its supporting information, or deposited to a public repository. For example, in addition to summary statistics, the data points behind means, medians and variance measures should be available. If there are restrictions on publicly sharing data—e.g. participant privacy or use of data from a third party—those must be specified.

Reviewer #4: Yes

**********

5. Is the manuscript presented in an intelligible fashion and written in standard English?

PLOS ONE does not copyedit accepted manuscripts, so the language in submitted articles must be clear, correct, and unambiguous. Any typographical or grammatical errors should be corrected at revision, so please note any specific errors here.

Reviewer #4: Yes

**********

6. Review Comments to the Author

Please use the space provided to explain your answers to the questions above. You may also include additional comments for the author, including concerns about dual publication, research ethics, or publication ethics. (Please upload your review as an attachment if it exceeds 20,000 characters)

Reviewer #4: Line 173: A total of 1514 + 1202 is more than the total of 3708 patients who participated in the smoking cessation program.

Lines from 181 to 184 are repeated.

**********

7. PLOS authors have the option to publish the peer review history of their article (what does this mean?). If published, this will include your full peer review and any attached files.

If you choose “no”, your identity will remain anonymous but your review may still be made public.

**Do you want your identity to be public for this peer review?**  For information about this choice, including consent withdrawal, please see our Privacy Policy.

Reviewer #4: **Yes:** ABDULRAZZAQ YAHYA AHMED AL-KHAZZAN

**********

[NOTE: If reviewer comments were submitted as an attachment file, they will be attached to this email and accessible via the submission site. Please log into your account, locate the manuscript record, and check for the action link "View Attachments". If this link does not appear, there are no attachment files.]

To ensure your figures meet our technical requirements, please review our figure guidelines: https://journals.plos.org/plosone/s/figures

You may also use PLOS’s free figure tool, NAAS, to help you prepare publication quality figures: https://journals.plos.org/plosone/s/figures#loc-tools-for-figure-preparation.

NAAS will assess whether your figures meet our technical requirements by comparing each figure against our figure specifications.

# Author comment: Author response to Decision Letter 4

**DOI:** 10.1371/journal.pone.0331514.r008

2 May 2026

6. Review Comments to the Author

Please use the space provided to explain your answers to the questions above. You may also include additional comments for the author, including concerns about dual publication, research ethics, or publication ethics. (Please upload your review as an attachment if it exceeds 20,000 characters)

Reviewer #4: Line 173: A total of 1514 + 1202 is more than the total of 3708 patients who participated in the smoking cessation program.

Lines from 181 to 184 are repeated.

→Thank you for the comments. As you pointed out, the numbers in the text were incorrect and, lines from 181 to 184 were repeated. Therefore, we have made the necessary corrections to the points you mentioned.

Attachment Submitted filename: Response to Reviewers.(4) docx.docx

# Editor report: Decision Letter 4

**DOI:** 10.1371/journal.pone.0331514.r009

**Authors:** Mukhtiar Baig

7 Jul 2026

Enhancing the long-term abstinence rate of smoking through telemedicine: A multicenter, retrospective cohort study in Japan

PONE-D-25-20021R4

Dear Dr. Takakura,

We’re pleased to inform you that your manuscript has been judged scientifically suitable for publication and will be formally accepted for publication once it meets all outstanding technical requirements.

Within one week, you’ll receive an e-mail detailing the required amendments. When these have been addressed, you’ll receive a formal acceptance letter and your manuscript will be scheduled for publication.

An invoice will be generated when your article is formally accepted. Please note, if your institution has a publishing partnership with PLOS and your article meets the relevant criteria, all or part of your publication costs will be covered. Please make sure your user information is up-to-date by logging into Editorial Manager at Editorial Manager® and clicking the ‘Update My Information' link at the top of the page. For questions related to billing, please contact billing support.

If your institution or institutions have a press office, please notify them about your upcoming paper to help maximize its impact. If they’ll be preparing press materials, please inform our press team as soon as possible -- no later than 48 hours after receiving the formal acceptance. Your manuscript will remain under strict press embargo until 2 pm Eastern Time on the date of publication. For more information, please contact onepress@plos.org.

Kind regards,

Mukhtiar Baig, Ph.D.

Academic Editor

PLOS One

# Editor report: Acceptance letter

**DOI:** 10.1371/journal.pone.0331514.r010

**Authors:** Mukhtiar Baig

PONE-D-25-20021R4

PLOS One

Dear Dr. Takakura,

I'm pleased to inform you that your manuscript has been deemed suitable for publication in PLOS One. Congratulations! Your manuscript is now being handed over to our production team.

At this stage, our production department will prepare your paper for publication. This includes ensuring the following:

* All references, tables, and figures are properly cited

* All relevant supporting information is included in the manuscript submission,

* There are no issues that prevent the paper from being properly typeset

You will receive further instructions from the production team, including instructions on how to review your proof when it is ready. Please keep in mind that we are working through a large volume of accepted articles, so please give us a few days to review your paper and let you know the next and final steps.

Lastly, if your institution or institutions have a press office, please let them know about your upcoming paper now to help maximize its impact. If they'll be preparing press materials, please inform our press team within the next 48 hours. Your manuscript will remain under strict press embargo until 2 pm Eastern Time on the date of publication. For more information, please contact onepress@plos.org.

You will receive an invoice from PLOS for your publication fee after your manuscript has reached the completed accept phase. If you receive an email requesting payment before acceptance or for any other service, this may be a phishing scheme. Learn how to identify phishing emails and protect your accounts at https://explore.plos.org/phishing.

If we can help with anything else, please email us at customercare@plos.org.

Thank you for submitting your work to PLOS ONE and supporting open access.

Kind regards,

PLOS ONE Editorial Office Staff

on behalf of

Professor Mukhtiar Baig

Academic Editor

PLOS One
